# CYFIP1 Coordinates mRNA Translation and Cytoskeleton Remodeling to Ensure Proper Dendritic Spine Formation

**DOI:** 10.1016/j.neuron.2013.06.039

**Published:** 2013-09-18

**Authors:** Silvia De Rubeis, Emanuela Pasciuto, Ka Wan Li, Esperanza Fernández, Daniele Di Marino, Andrea Buzzi, Linnaea E. Ostroff, Eric Klann, Fried J.T. Zwartkruis, Noboru H. Komiyama, Seth G.N. Grant, Christel Poujol, Daniel Choquet, Tilmann Achsel, Danielle Posthuma, August B. Smit, Claudia Bagni

**Affiliations:** 1 VIB Center for Biology of Disease, KULeuven, 3000 Leuven, Belgium; 2 Center for Human Genetics and Leuven Institute for Neuroscience and Disease (LIND), KULeuven, 3000 Leuven, Belgium; 3 Department of Molecular and Cellular Neurobiology, Center for Neurogenomics and Cognitive Research, Neuroscience Campus Amsterdam, VU University Medical Center Amsterdam, 1081 HV Amsterdam, The Netherlands; 4 Department of Functional Genomics, Center for Neurogenomics and Cognitive Research, Neuroscience Campus Amsterdam, VU University Medical Center Amsterdam, 1081 HV Amsterdam, The Netherlands; 5 Department of Clinical Genetics, Center for Neurogenomics and Cognitive Research, Neuroscience Campus Amsterdam, VU University Medical Center Amsterdam, 1081 HV Amsterdam, The Netherlands; 6 Center for Neural Science, New York University, New York, NY 10003, USA; 7 Molecular Cancer Research, Center for Biomedical Genetics and Cancer Genomics Center, University Medical Center Utrecht, 3584 CG Utrecht; 8 Centre for Clinical Brain Sciences and Centre for Neuroregeneration, University of Edinburgh, Edinburgh EH16 4SB, UK; 9 CNRS, Bordeaux Imaging Center, UMS 3420, 33000 Bordeaux, France; 10 University of Bordeaux, UMS 3420, 33077, Bordeaux, France; 11 CNRS, Interdisciplinary Institute for Neuroscience, IINS, UMR 5297, 33000 Bordeaux, France; 12 University of Bordeaux, UMR 5297, 33077, Bordeaux, France; 13 Department of Child and Adolescent Psychiatry, Erasmus University Medical Center/Sophia Child Hospital, 3000 CB Rotterdam, The Netherlands; 14 Department of Biomedicine and Prevention, University “Tor Vergata,” 00133 Rome, Italy

## Abstract

The *CYFIP1/SRA1* gene is located in a chromosomal region linked to various neurological disorders, including intellectual disability, autism, and schizophrenia. CYFIP1 plays a dual role in two apparently unrelated processes, inhibiting local protein synthesis and favoring actin remodeling. Here, we show that brain-derived neurotrophic factor (BDNF)-driven synaptic signaling releases CYFIP1 from the translational inhibitory complex, triggering translation of target mRNAs and shifting CYFIP1 into the WAVE regulatory complex. Active Rac1 alters the CYFIP1 conformation, as demonstrated by intramolecular FRET, and is key in changing the equilibrium of the two complexes. CYFIP1 thus orchestrates the two molecular cascades, protein translation and actin polymerization, each of which is necessary for correct spine morphology in neurons. The CYFIP1 interactome reveals many interactors associated with brain disorders, opening new perspectives to define regulatory pathways shared by neurological disabilities characterized by spine dysmorphogenesis.

## Highlights

• BDNF-Rac1 signaling redistributes CYFIP1 between eIF4E and WRC
• FRET demonstrates that BDNF-Rac1 signaling alters CYFIP1 conformation
• CYFIP1-eIF4E-WRC regulates ARC synthesis, actin polymerization, and spine morphology
• The CYFIP1 interactome is enriched in genes implicated in neurological disorders

## Teaser

CYFIP1 plays a dual role in inhibiting local protein synthesis and favoring actin remodeling. De Rubeis et al. show that BDNF-driven synaptic signaling releases CYFIP1 from the translational inhibitory complex, triggering translation of target mRNAs and shifting CYFIP1 into the WAVE regulatory complex.

## Introduction

Genetic alterations of the pathways controlling local protein synthesis in neurons contribute to diverse intellectual disabilities (ID) and autism spectrum disorders (ASDs) (Ehninger and Silva, 2009). These disorders are synaptopathies (Ehninger and Silva, 2009) in which dysgenesis of dendritic spines is a recurrent anatomical feature (Penzes et al., 2011). Fragile X syndrome (FXS) is the most common form of inherited ID and a frequent monogenic cause of ASD (Belmonte and Bourgeron, 2006, Hatton et al., 2006, Jacquemont et al., 2007, Turk, 2011). Patients with FXS display dendritic spine defects (Irwin et al., 2001), neurodevelopmental delay, and autistic-like phenotype (Jacquemont et al., 2007). FXS is due to loss of function of the RNA-binding protein FMRP (Bagni et al., 2012, Bassell and Warren, 2008), which regulates dendritic targeting of mRNAs (Dictenberg et al., 2008) and controls protein synthesis and mRNA decay in neuronal soma and at synapses (Bassell and Warren, 2008). High-throughput screenings (Brown et al., 2001, Darnell et al., 2011, Klemmer et al., 2011, Liao et al., 2008, Miyashiro et al., 2003) have revealed that a wide array of neuronal mRNAs is targeted by FMRP, suggesting that simultaneous dysregulation of many proteins contributes to FXS.

A key functional partner of FMRP is the cytoplasmic FMRP-interacting protein 1, CYFIP1 (Napoli et al., 2008, Schenck et al., 2003, Schenck et al., 2001) also known as “specific Rac1-activated” (SRA1) protein (Kobayashi et al., 1998). *CYFIP1* is located within a hot spot for ASD (chr15q11.2), close to a region critical for two ASD-related syndromes: the Angelman and Prader-Willi syndromes. Microdeletions or microduplications of the region, including *CYFIP1* and three other genes, cosegregate with cognitive disabilities and ASD (Cooper et al., 2011, Doornbos et al., 2009, Leblond et al., 2012, Nishimura et al., 2007, van der Zwaag et al., 2010, von der Lippe et al., 2010). *CYFIP1* messenger RNA (mRNA) is downregulated in a subgroup of FXS patients who have the Prader-Willi phenotype and show severe ASD and obsessive-compulsive behavior (Nowicki et al., 2007). In addition, *CYFIP1* has recently been linked to schizophrenia (SCZ) (Tam et al., 2010, Zhao et al., 2012).

Together with FMRP, CYFIP1 represses neuronal protein synthesis: FMRP tethers specific mRNAs to CYFIP1, which in turn sequesters the cap-binding protein eIF4E, thereby preventing initiation of translation (Napoli et al., 2008). Upon activation of the brain-derived neurotrophic factor (BDNF)/NT-3 growth factor receptor (TrkB) or group I metabotropic glutamate receptors (mGluRs), CYFIP1 is released from eIF4E and translation ensues (Napoli et al., 2008). Furthermore, CYFIP1 is part of the Wave Regulatory Complex (WRC), a heteropentamer containing also WAVE1/2/3, ABI1/2, NCKAP1 and HPSC300 (Takenawa and Suetsugu, 2007). The WRC regulates the actin-nucleating activity of the Arp2/3 complex and it can be activated through the small GTPase Rac1, kinases, and phospholipids (Chen et al., 2010, Eden et al., 2002, Lebensohn and Kirschner, 2009). In particular, the Rac1 signaling can activate the WRC through CYFIP1 (Chen et al., 2010, Eden et al., 2002, Kobayashi et al., 1998, Schenck et al., 2003, Steffen et al., 2004). Rearrangements of the actin cytoskeleton strongly influence the formation, retraction, motility, stability, and shape of the dendritic spines (Tada and Sheng, 2006), and genetic ablation of WRC components affects spine morphology and excitability (Grove et al., 2004, Kim et al., 2006, Soderling et al., 2007, Wiens et al., 2005). However, the interplay of this process with other events regulating spine function, such as local translation, is still unknown.

Here, we demonstrate that active Rac1 changes the equilibrium between two distinct CYFIP1 complexes, activating the translation of mRNAs important for synaptic structure and function, such as *Arc/Arg3.1* mRNA. This switch occurs through a conformational change in CYFIP1, detectable by Förster resonance energy transfer (FRET). Knockdown of *Cyfip1* or mutations in the regions interacting with eIF4E or WRC produce dendritic spine defects resembling those found in FXS and other synaptopathies. These findings shed light on the molecular mechanisms that tune the balance between translational control and actin remodeling at synapses. The identification of interaction partners of CYFIP1 suggests that neurological disorders characterized by spine dysmorphogenesis might be due to perturbations in the balance between these two CYFIP1 interconnected pathways.

## Results

### CYFIP1 Is Part of Two Complexes

**Figure 1:**
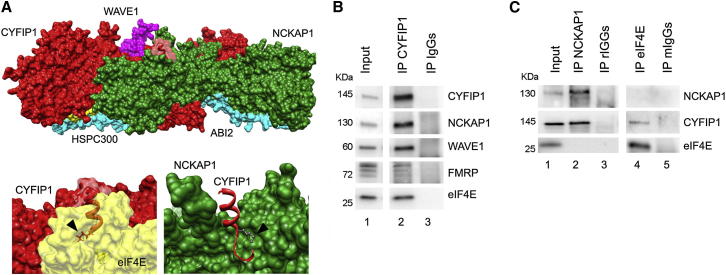
CYFIP1 Participates in Two Distinct Complexes (A) Analysis of the crystal structure of the WRC. Upper panel: the eIF4E-binding site (light red, aa 733–751 of the human protein) of CYFIP1 (red) is partially occluded by NCKAP1 (green). Lower panels: left, a detail showing the interaction between CYFIP1 (red) and eIF4E (yellow); right, a detail showing that Lys743 is covered by NCKAP1 (green) when CYFIP1 (red) is within the WRC. Lys743 that is crucial for the interaction with eIF4E is highlighted with an arrowhead in both panels. (B) CYFIP1 IP from synaptoneurosomes. Lane 1, input (1/100); lane 2, CYFIP1 IP; lane 3, control IP (rabbit IgGs). Lanes shown belong to the same blot, n = 6. (C) NCKAP1 and eIF4E IPs from synaptoneurosomes. Lane 1, input (1/100); lane 2, NCKAP1 IP; lane 3, control (rIgGs) IP; lane 4, eIF4E IP; lane 5, control (mIgGs) IP. Lanes shown belong to the same blot, n = 3. See also Figure S1 and Table S1.

To dissect the CYFIP1 function and its possible crosstalk with the FMRP-eIF4E translational complex and the actin-regulatory complex WRC, we investigated the structural organization of the two CYFIP1 complexes. According to the crystal structure of the WRC that includes CYFIP1 (Chen et al., 2010), NCKAP1 interacts with CYFIP1 over a large surface (Figure 1A, upper panel); the lysine critical for the binding to eIF4E (Lys743) (Napoli et al., 2008) is covered by NCKAP1 and therefore is not accessible to solvent when CYFIP1 is in the WRC (Figure 1A, bottom panels, Table S1). These structural data indicate that the same CYFIP1 molecule cannot simultaneously interact with the WRC and eIF4E.

Synapses are severely affected in FXS and other neurological disorders (Fiala et al., 2002, Penzes et al., 2011, Valnegri et al., 2012). Electron microscopy (EM) and biochemical studies revealed that CYFIP1, at synapses, is enriched in postsynaptic compartments (Figure S1 available online). In mouse cortical synaptoneurosomes, CYFIP1 coimmunoprecipitates with FMRP, eIF4E, NCKAP1, and WAVE1 (Figure 1B). Furthermore, immunoprecipitation of NCKAP1 revealed the presence of CYFIP1 but not eIF4E, whereas immunoprecipitation of the eIF4E complex detected CYFIP1 but not NCKAP1 (Figure 1C). We conclude that CYFIP1 engages in two distinct complexes.

### Synaptic Stimulation Changes CYFIP1 Distribution between the Two Molecular Complexes

**Figure 2:**
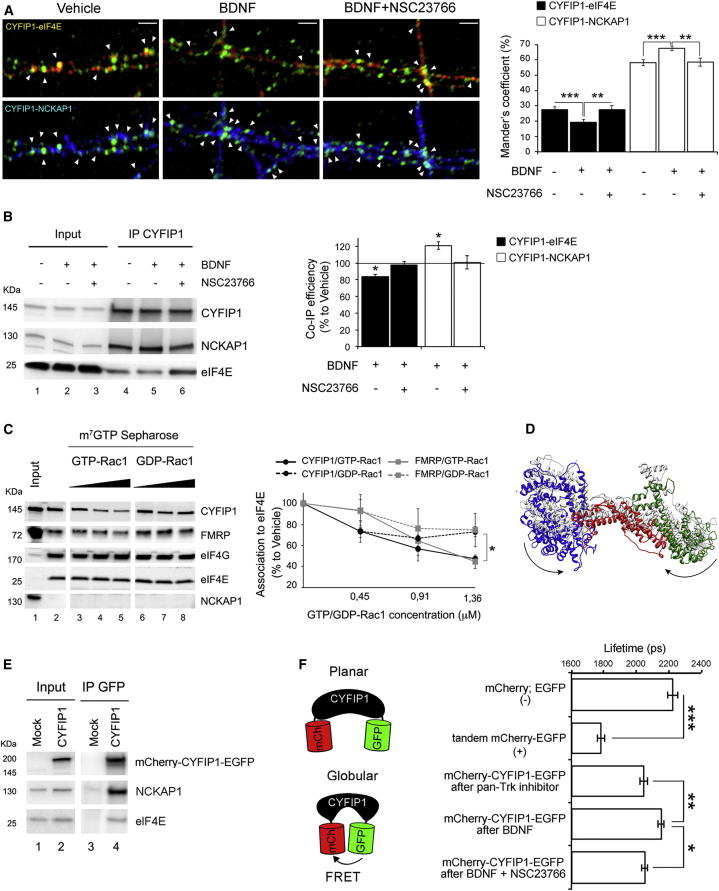
Upon BDNF Treatment CYFIP1 Shifts between eIF4E and WRC through a Conformational Change Induced by Active Rac1 (A) BDNF changes CYFIP1-eIF4E and CYFIP1-NCKAP1 colocalization in a Rac1-dependent manner. Left: representative dendrites costained for CYFIP1-eIF4E (yellow, upper row) and CYFIP1-NCKAP1 (cyan, lower row; scale bar represents 5 μm). See Figure S2B for single staining (green, CYFIP1; red, eIF4E; blue, NCKAP1). Neurons treated with vehicle or BDNF (100 ng/ml for 30 min) with/without NSC23766 (200 μM for 10 min pretreatment) are shown. Right: percentage of overlap expressed by Mander’s coefficient for CYFIP1-eIF4E (black) and CYFIP1-NCKAP1 (white) (three dendrites from at least ten neurons/condition; one-way ANOVA) with Bonferroni post hoc test; ^∗∗^p < 0.01, ^∗∗∗^p < 0.001). Arrowheads indicate colocalization puncta. Bars represent mean ± SEM. (B) BDNF changes CYFIP1-eIF4E and CYFIP1-NCKAP1 interactions through Rac1 activation. Left: CYFIP1 IP from synaptoneurosomes stimulated with BDNF (100 ng/ml for 30 min) with/without NSC23766 (200 μM for 10 min pretreatment). Lane 1, inputs (1/100) for vehicle; lane 2, BDNF; lane 3, BDNF + NSC23766; lanes 4–6, IPs for vehicle, BDNF and BDNF + NSC23766. Right, histogram represents CYFIP1-eIF4E (black) and CYFIP1-NCKAP1 (white) coimmunoprecipitation relative to untreated, control samples (n = 4, paired Student’s t test, ^∗^p < 0.05). Bars represent mean ± SEM. (C) Active Rac1 reduces the affinity of CYFIP1 for eIF4E. Left panel: m^7^GTP chromatography on cortical extracts after incubation with exogenous GTP-Rac1 or GDP-Rac1. Lane 1, input (1/100); lane 2, m^7^GTP chromatography; lanes 3–5, m^7^GTP chromatography in the presence of 0.45, 0.91, or 1.36 μM of GTP-Rac1, respectively; lanes 6–8, as lanes 3–5 but with GDP–Rac1. Right panel: association of CYFIP1 (black) and FMRP (gray) to eIF4E as percentage to vehicle (n = 6; repeated-measures ANOVA, ^∗^p < 0.05). Bars represent mean ± SEM. (D) Molecular dynamics simulation of CYFIP1 predicts an alternative conformation. Superposition of the CYFIP1 X-ray structure in the WRC (“planar,” light gray) upon the CYFIP1 structure obtained by clustering the conformations that dominate the end of the 135-ns molecular dynamics simulation (more “globular,” colored). The N-terminal, central, and C-terminal domains of the simulated structure are shown in blue, red, and green, respectively. The arrows indicate the principal movements during the conformational change. (E) mCherry-CYFIP1-EGFP coimmunoprecipitates with NCKAP1 and eIF4E. CYFIP1 was immunoprecipitated with an anti-GFP antibody. Lane 1, mock input (1/100); lane 2, transfected cells input (1/100); lane 3, IP from mock transfected cells; lane 4, IP from transfected cells. (F) Left: sketch showing how FRET reveals a globular CYFIP1 conformation. Right: in neurons CYFIP1 exists in a globular conformation that changes after BDNF treatment. Top to bottom: free EGFP and free mCherry (negative control); tandem mCherry-EGFP (intramolecular FRET); mCherry-CYFIP1-EGFP in neurons treated with the pan-Trk inhibitor k252a (100 nM for 24 hr); mCherry-CYFIP1-EGFP after BDNF (100 ng/ml for 30 min) treatment; mCherry-CYFIP1-EGFP after Rac1 inhibitor NSC23766 (200 μM for 10 min pretreatment) followed by BDNF treatment. At least n = 30, one-way ANOVA with Bonferroni post hoc test, ^∗^p < 0.05, ^∗∗^p < 0.01, ^∗∗∗^p < 0.001. Bars represent mean ± SEM. See also Figures S2 and S3.

Synaptic activity leads to an increase of protein synthesis as well as actin remodeling (Bramham, 2008). Given the presence of CYFIP1 in the FMRP-eIF4E translational complex and the actin-regulatory complex WRC, we investigated whether its distribution over these two complexes might change after synaptic stimulation. Therefore, we stimulated cortical neurons with BDNF at 15 days in vitro (DIV) (Figure S2A), a stage when FMRP, CYFIP1, and eIF4E are highly expressed and neurons are mature (Figure S2A). We stimulated neurons with BDNF, which induces translation (Aakalu et al., 2001, Schratt et al., 2004, Takei et al., 2004) and actin remodeling (Bramham, 2008), and followed the subsequent changes in the colocalization of CYFIP1 with eIF4E or NCKAP1. Stimulation by BDNF significantly reduced the degree of CYFIP1-eIF4E colocalization, and concomitantly increased the number of CYFIP1-NCKAP1 puncta, suggesting that CYFIP1 distribution changes between these complexes upon TrkB receptor activation (Figures 2A and S2B). The magnitude of these changes is similar to those observed with manipulations that alter interactions of eIF4E with canonical eIF4E-BPs (Costa-Mattioli et al., 2009, Richter and Klann, 2009, Sonenberg and Hinnebusch, 2009). These changes were observed 15 min after BDNF stimulation (Figure S2C). Only a very small proportion of CYFIP1 remained not engaged within these two complexes (∼15% according to the colocalization data). Consistently, blue native PAGE (BN-PAGE) revealed that the majority, if not all, of CYFIP1 is part of high molecular weight complexes (Figure S2D). Based on these data, we infer that a “free” CYFIP1 pool is minor.

We then aimed at identifying the factors regulating this equilibrium. A candidate is Rac1, because in its active form (GTP-Rac1), it interacts with CYFIP1 (Kobayashi et al., 1998) and favors WRC activation (Chen et al., 2010, Eden et al., 2002, Schenck et al., 2003, Steffen et al., 2004). To test this hypothesis, we used NSC23766, a specific inhibitor of Rac1 activation (Gao et al., 2004) (Figure S2E). Addition of NSC23766 before BDNF stimulation prevented the redistribution of CYFIP1 (Figure 2A), indicating that active Rac1 is needed for the effect of BDNF on the CYFIP1 complexes. To further monitor the dynamics of CYFIP1 redistribution, we quantified the changes in fluorescence of EYFP-CYFIP1, Cerulean-NCKAP1, and eIF4E-mCherry in spines of BDNF-stimulated primary neurons over time (Figure S3). We observed that the ratio of Cerulean-NCKAP1 over EYFP-CYFIP1 steadily increases, indicating a build-up of WRC (Figure S3C).

CYFIP1 redistribution between eIF4E- and NCKAP1-containing complexes was further corroborated by biochemical evidence in isolated synaptoneurosomes: BDNF stimulation increased the amount of CYFIP1 coprecipitating with NCKAP1, and conversely reduced its binding to eIF4E; the Rac1 inhibitor was able to prevent the CYFIP1 redistribution (Figure 2B).

To investigate whether active Rac1 directly changes the ability of CYFIP1 to bind eIF4E, we used GTP-Rac1 as exogenous competitor in m^7^GTP chromatography on cortical lysates. Indeed, increasing concentrations of GTP-Rac1 reduced the degree of binding of CYFIP1 to eIF4E, whereas inactive Rac1 (GDP-Rac1) had no effect (Figure 2C). The association of FMRP to eIF4E was also reduced, whereas no changes were observed for eIF4G. NCKAP1 did not copurify at all with eIF4E, showing that the assay specifically allowed isolation of eIF4E-associated complexes. These data indicate that exogenous active Rac1 partially dissolves a preassembled CYFIP1-eIF4E complex. To address whether Rac1 also drives the distribution of CYFIP1 over the two complexes in other physiological and cellular contexts, we monitored the CYFIP1-eIF4E complex upon serum restoration in serum-deprived HEK293T cells (Figure S4A). In agreement with our findings in brain, CYFIP1 and FMRP were rapidly released from eIF4E upon addition of serum, and then slowly reassociated (Figure S4B), whereas Rac1 inhibitor abolished the release of the translational inhibitory complex (Figure S4C).

Finally, we investigated how active Rac1 changes the binding affinity of CYFIP1 for eIF4E and thereby favors the association of CYFIP1 with the WRC. A possibility is that CYFIP1 exists in two different conformations, and that GTP-Rac1 triggers a transition between the two. The crystal structure of the WRC showed that CYFIP1 has a planar conformation (Chen et al., 2010). We extracted CYFIP1 from the WRC and let it evolve in a molecular dynamics simulation for 135 ns. We obtained a CYFIP1 molecule with a predicted more “globular” conformation and a reduced distance between the N and C termini (∼7 nm instead of 12.8 nm measured for CYFIP1 in the WRC crystal structure) (Figure 2D). The consequence of this conformational change is that the domain carrying the eIF4E-binding site moves toward the outside (Figure 2D), allowing Lys743 to interact with Glu132 of eIF4E (Figure 1A) (Napoli et al., 2008). To validate the predicted second CYFIP1 conformation, we applied intramolecular FRET on HEK293T cells transfected with a CYFIP1 harboring mCherry and EGFP at its N and C termini (mCherry-CYFIP1-EGFP) (Figure 2E). The presence of two fluorescent tags did not inhibit the interaction of CYFIP1 with eIF4E and NCKAP1 (Figure 2E). FRET was revealed by measuring the donor’s fluorescence lifetime (for details, see legend to Figure S4D). Only the globular conformation might result in FRET, due to a distance between the termini of ∼7 nm, whereas the separation of 12.8 nm in the planar conformation would not allow substantial Förster-type resonance (R_0_ = ∼5 nm) (Albertazzi et al., 2009). mCherry-CYFIP1-EGFP exhibited significant FRET, indicating that CYFIP1 exists in a conformation where the two fluorophores are within range for a Förster-type interaction. Inhibition of Rac1 activation by NSC23766 further increased the FRET signal, which is most likely explained by a higher number of molecules in the more globular conformation, the conformation that allows CYFIP1 to bind eIF4E. Importantly, these data were confirmed and further extended in primary cortical neurons (Figure 2F). To promote the engagement of mCherry-CYFIP1-EGFP in the translation inhibitory complexes, we treated primary neurons with the panTrk inhibitor k252a (Petroulakis and Wang, 2002). As expected, such treatment decreased ARC synthesis and eIF4E phosphorylation (Gingras et al., 1999) (Figure S4E). Under these conditions, a significant FRET was detected in neurons transfected with mCherry-CYFIP1-EGFP. This shows that also in neurons a subpopulation of CYFIP1 molecules exists in a more globular conformation. Treatment with BDNF attenuated the FRET signal, indicating that a fraction of CYFIP1 molecules switched to the planar conformation. The Rac1 inhibitor blocked the effects of BDNF and restored the equilibrium back to the more globular conformation. These data provide independent experimental support that the switch of CYFIP1 between the two complexes might be facilitated by a conformational change mediated by Rac1.

### Rac1 Affects the CYFIP1-FMRP Regulated mRNA Translation

**Figure 3:**
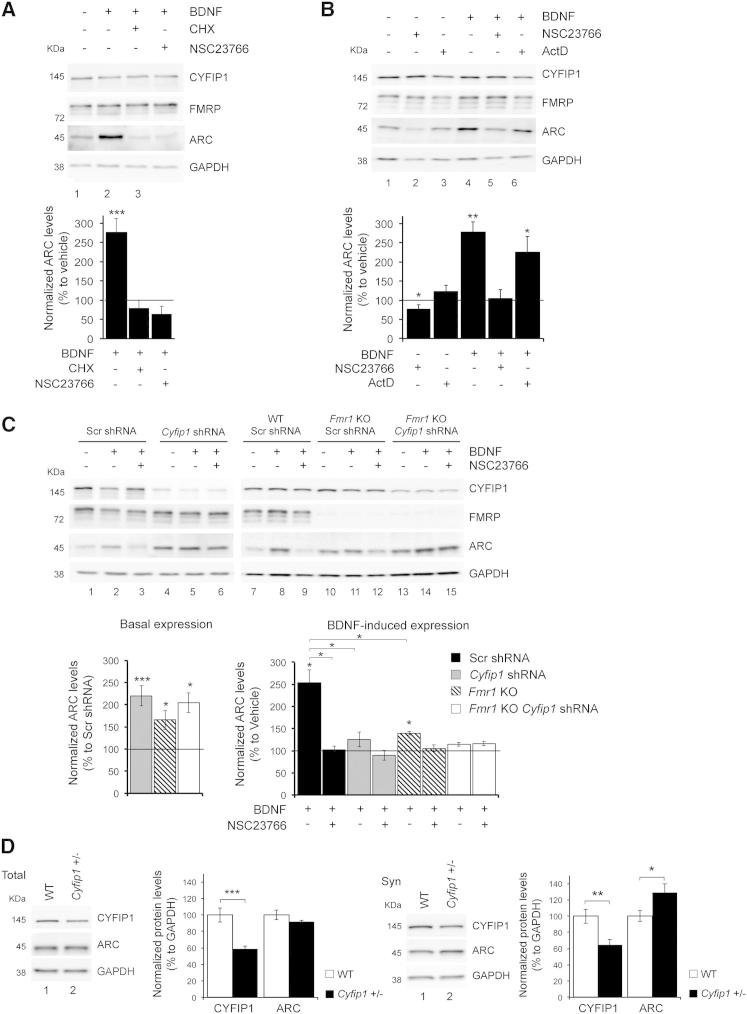
Rac1 Regulates CYFIP1 and FMRP-Dependent mRNA Translation (A) ARC is upregulated by BDNF in a Rac1-dependent manner. Upper panel: immunoblot analysis of cortical neurons stimulated with BDNF (100 ng/ml for 30 min) +/– cycloheximide (CHX, 60 μM for 30 min) or NSC23766 (200 μM for 10 min pretreatment). Lower panel: ARC protein levels normalized to GAPDH and expressed as percentage to vehicle (n = 4, one-way ANOVA with Holm’s post hoc test, ^∗∗∗^p < 0.001). Bars represent mean ± SEM. (B) Upregulation of ARC upon BDNF treatment is protein-synthesis-dependent. Upper panel: immunoblot analysis of cortical neurons stimulated with NSC23766 (200 μM for 20 min) or BDNF +/− pretreatment with actinomycin D (Act D) (1 μg/ml for 30 min) or NSC23766 (200 μM for 10 min) as indicated above the lanes. Lower panel: quantification of ARC protein levels normalized to GAPDH and expressed as percentage of vehicle control (n = 3, one-way ANOVA with Holm’s post hoc test, ^∗^p < 0.05, ^∗∗^p < 0.01). Bars represent mean ± SEM. (C) Synthesis of ARC is altered in *Cyfip1* knockdown, *Fmr1* KO, and *Fmr1* KO/*Cyfip1* knockdown neurons. Upper panel: immunoblot of cortical neurons stimulated with BDNF (100 ng/ml for 30 min) +/– NSC23766 (200 μM for 10 min pretreatment). Lanes 1–3, scrambled shRNA; lanes 4–6, *Cyfip1* shRNA, lanes 7–9, scrambled shRNA in WT neurons; lanes 10–12, scrambled shRNA in *Fmr1* KO neurons; lanes 13–15, *Cyfip1* shRNA in *Fmr1* KO neurons. Lower right panels: basal expression of CYFIP1, FMRP, and ARC levels in vehicle-treated neurons. Protein levels were normalized to GAPDH and shown as percentage of scrambled shRNA. Grey, *Cyfip1* shRNA; gray stripes, scrambled shRNA in *Fmr1* KO; white, *Cyfip1* shRNA in *Fmr1* KO (n = 6, one-way ANOVA with Holm’s post hoc correction, ^∗^p < 0.05, ^∗∗∗^p < 0.001). Lower right panel: activity-induced ARC expression. ARC levels after BDNF +/− NSC23766 expressed as percentage to vehicle-treated neurons. Black, scrambled shRNA; gray, *Cyfip1* shRNA; gray stripes, scrambled shRNA in *Fmr1* KO; white, *Cyfip1* shRNA in *Fmr1* KO (n = 6, two-way ANOVA with Holm’s post hoc correction, ^∗^p < 0.05). Bars represent mean ± SEM. (D) Left panels: ARC level is unaffected in total cortical extracts from *Cyfip1*^*+/−*^ mice. Immunoblot of CYFIP1, ARC, and GAPDH in WT (lane 1) and *Cyfip1*^*+/−*^ (lane 2) mice. Protein levels were normalized to GAPDH and shown as percentage of WT; white, WT (n = 5); black, *Cyfip1*^*+/−*^ n = 3, (Student’s t test, ^∗∗∗^p < 0.001). Right panels: as in the left panels but in synaptoneurosomes (n = 3, Student’s t test, ^∗^p < 0.05; ^∗∗^p < 0.01). Bars represent mean ± SEM.

Our findings indicate that Rac1 influences the switch of CYFIP1 from eIF4E to WRC, which predicts that it should also modulate the translation of CYFIP1-FMRP target mRNAs. To test this hypothesis, we examined the synthesis of the well-characterized FMRP target *Arc/Arg3.1* (Napoli et al., 2008, Niere et al., 2012, Park et al., 2008, Zalfa et al., 2003) in primary cortical neurons at DIV15. As shown in Figure 3, ARC expression was robustly induced by BDNF, and this effect was due to protein synthesis, because it was blocked by concomitant treatment with cycloheximide (inhibitor of protein synthesis; Figure 3A) but not by actinomycin D (inhibitor of transcription; Figure 3B). ARC synthesis triggered by BDNF was completely abolished by pretreatment with NSC23766 (Figures 3A and 3B). These effects were not due to interference with TrkB activation or its signaling cascade, because BDNF-induced TrkB and ERK1/2 phosphorylation was not affected by NSC23766 (Figure S4F), indicating that Rac1 inhibition does not disrupt primarily TrkB signaling. When prolonged activation of TrkB was blocked with Dynasore (a chlatrin-dependent endocytosis inhibitor), ARC levels were still induced by BDNF.

To finally demonstrate that Rac1 requires CYFIP1 and FMRP as downstream effectors to regulate ARC synthesis, *Cyfip1* knocked-down or *Fmr1* knock-out (KO) neurons were stimulated with BDNF with or without NSC23766. *Cyfip1* was knocked-down in cortical neurons (DIV9) with lentivirus carrying a “short hairpin” (sh) RNA directed against *Cyfip1* or a scrambled shRNA (i.e., an RNA hairpin with a random sequence). Three independent shRNAs were tested, and the shRNA with highest efficiency in knocking down *Cyfip1* (shRNA 319; Figure S5A) was used for subsequent experiments. We found that both CYFIP1 and FMRP affect basal and activity-induced ARC synthesis. When CYFIP1 expression was reduced to 16% (Figure S5A), ARC basal levels were significantly increased (Figure 3C). Moreover, ARC was robustly upregulated after BDNF treatment in control, but not in *Cyfip1*-silenced neurons; also the Rac1 inhibitor NSC23766 did not affect ARC levels in CYFIP1-deficient cells (Figure 3C). Similarly, loss of FMRP increased ARC basal expression (Figure 3C). Furthermore, ARC synthesis triggered by BDNF was much lower in *Fmr1* KO neurons compared with wild-type (WT); inhibition of Rac1 activation before BDNF stimulation blocked ARC synthesis in WT as well as the residual synthesis in *Fmr1* KO neurons, whereas no effect was observed in *Cyfip1*-silenced neurons (Figure 3C). *Fmr1* KO neurons silenced for *Cyfip1* phenocopied CYFIP1-deficient neurons, further confirming that FMRP and CYFIP1 act in the same pathway (Figure 3C).

We also investigated ARC levels in mice where CYFIP1 expression was genetically reduced. Because *Cyfip1* KO animals are embryonic-lethal (our observation and Bozdagi et al., 2012), we used heterozygous animals where CYFIP1 levels are reduced by 40% (Figure 3D). We examined ARC expression in both total brain cortex and cortical synaptoneurosomes and found that *Cyfip1*^+/−^ mice have elevated ARC levels at synapses (Figure 3D).

These data support the hypothesis that FMRP and CYFIP1 regulate protein synthesis downstream of Rac1 activation. Activated Rac1 reshapes the CYFIP1-eIF4E complex through a conformational change, so that when translation inhibition is lifted, more CYFIP1 becomes available for the WRC.

### CYFIP1 Affects Spine Morphology

**Figure 4:**
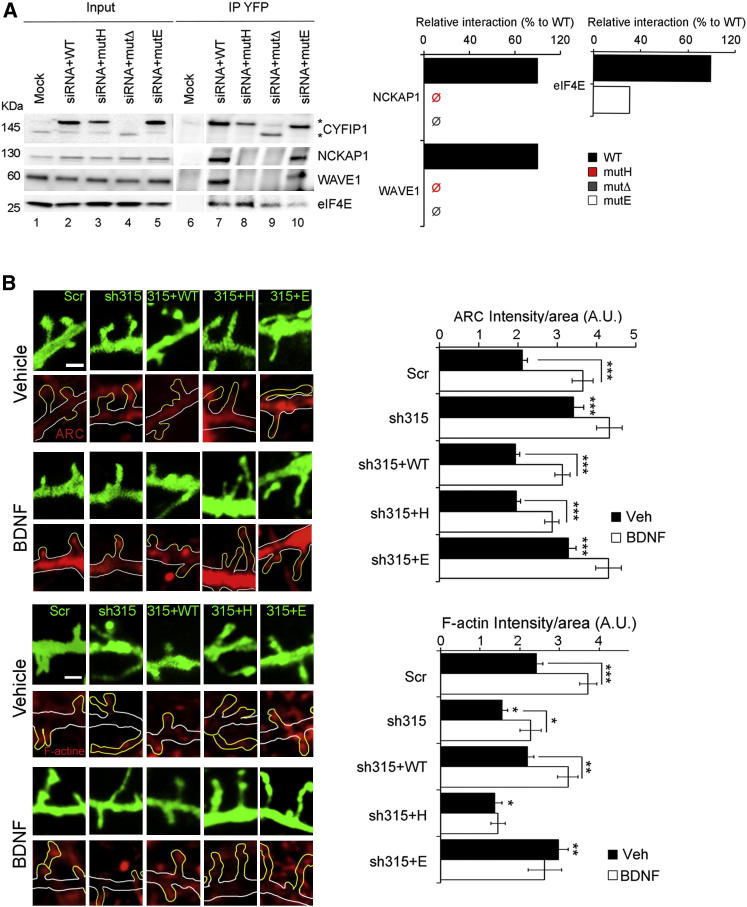
CYFIP1 Deficiency or Mutations Affecting Interaction with eIF4E or NCKAP1 Alter Synaptic ARC and F-Actin (A) Dissection of the CYFIP1 interactions with NCKAP1 (mutΔ and mutH) or with eIF4E (mutE). Left panel: IP for YFP-CYFIP1 WT or mutants in HEK293T cells silenced for endogenous *CYFIP1*. Lane 1, input (1/50) from mock-transfected cells; lanes 2–5, input *CYFIP1* siRNA with RNAi-resistant CYFIP1 WT, mutH, mutΔ, or mutE (1/50); lane 6, YFP-IP with mock-transfected cells; lanes 7–10, YFP-IP for WT, mutH, mutΔ, or mutE-CYFIP1. Asterisks indicate exogenous CYFIP1. Central panel: quantification of CYFIP1-NCKAP1 and CYFIP1-WAVE1 as percentage of WT (black) for mutH (red) and mutΔ (gray). Right panel: quantification of CYFIP1-eIF4E as percentage of WT (black) for mutE (white), see also Napoli et al. (2008). (B) Upper panels: CYFIP1 deficiency or mutations affecting the interaction with eIF4E or NCKAP1 alter synaptic ARC and F-actin levels. Panels show representative dendritic sections transfected with scrambled or *Cyfip1* shRNA (F-GFP, upper panels) and stained for ARC (red, lower panels) in vehicle or BDNF-treated neurons. Spines are highlighted in yellow. Scale bar represents 1 μm. Histogram represents the ARC immunosignal normalized to the spine area for neurons transfected as indicated on the x axis and treated with vehicle (black) or BDNF (white) (at least n = 150, two-way ANOVA with Holm’s post hoc test, ^∗∗∗^p < 0.001). Lower panels: as above for F-actin (at least n = 50, two-way ANOVA with Holm’s post hoc test, ^∗^p < 0.05, ^∗∗^p < 0.01, ^∗∗∗^p < 0.001). Bars represent mean ± SEM. See also Figure S4.

Our results suggest that CYFIP1 complexes have a specific function in synaptic protein synthesis and actin polymerization. As proof of principle, we aimed at uncoupling the two complexes and studying their contribution to protein translation and actin polymerization. For this purpose, we designed specific CYFIP1 mutants impairing the interactions with either eIF4E or NCKAP1. To reduce the CYFIP1-eIF4E interaction, we used a mutant replacing Lys743 with a Glu (mutant E), which has been shown to reduce the interaction with eIF4E (Napoli et al., 2008). To interfere with the CYFIP1-NCKAP1 complex, we studied the large surface of interaction between the two proteins (Chen et al., 2010), and found two hydrophobic patches on CYFIP1 that fit to corresponding sites on NCKAP1 (Figure S5B). The second patch shows a higher complementarity to NCKAP1, in particular in a stretch of eight consecutive hydrophobic amino acids (Ala1003–Ile1010), which was predicted as an essential binding site for NCKAP1. We therefore designed two mutants: mutant Δ, lacking the C-terminal domain that harbors the hydrophobic patch (aa 922–1251), and mutant H, in which the eight hydrophobic residues were replaced by glycines. WT and mutant proteins tagged with the yellow fluorescent protein (EYFP) were expressed in HEK293T cells (Figure S5C) and displayed correct cytoplasmic localization (data not shown). To promote the incorporation of the exogenous proteins into functional complexes, we silenced the endogenous *Cyfip1* with siRNAs directed against its 3′UTR (Figures 4A and S5D). Both mutant Δ and mutant H lost their affinity for NCKAP1 and consequently for WAVE1, but not for eIF4E, whereas the interaction with eIF4E was largely decreased with mutant E (Figure 4A), leaving unaffected the binding to NCKAP1 and WAVE1.

To study the contribution of the two CYFIP1 complexes on ARC synthesis and actin cytoskeleton at synapses, primary cortical neurons (DIV9) were transfected with scrambled or *Cyfip1* (sh315) shRNA, in combination with CYFIP1 WT, mutant H (affecting actin polymerization), or mutant E (affecting mRNA translation). ARC and F-actin were detected by immunolabeling in neurons at DIV14 with or without BDNF treatment, and the immunosignal was quantified in spines outlined by the membrane-targeted farnesylated GFP (F-GFP) carried by the shRNA construct. We found that CYFIP1 downregulation caused augmented ARC synthesis and reduced F-actin levels in spines (Figure 4B). Moreover, ARC and F-actin were enhanced after BDNF treatment, but not in *Cyfip1*-silenced neurons (Figure 4B). Cotransfection of the construct carrying CYFIP1 WT rescued all defects, both basal and BDNF-induced. As predicted, basal and inducible ARC expression was restored by mutant H, but not by mutant E. F-actin levels, in contrast, were rescued by mutant E, but not by mutant H. The fact that mutant E rescued F-actin expression but remains insensitive to BDNF stimulation (Figure 4B) might suggest that this pathway requires local translation in addition to WRC activation. In conclusion, the data demonstrate that the CYFIP1 mutants are valuable in separating the two functions of CYFIP1 in the regulation of local protein translation and the control of actin cytoskeleton at synapses.

**Figure 5:**
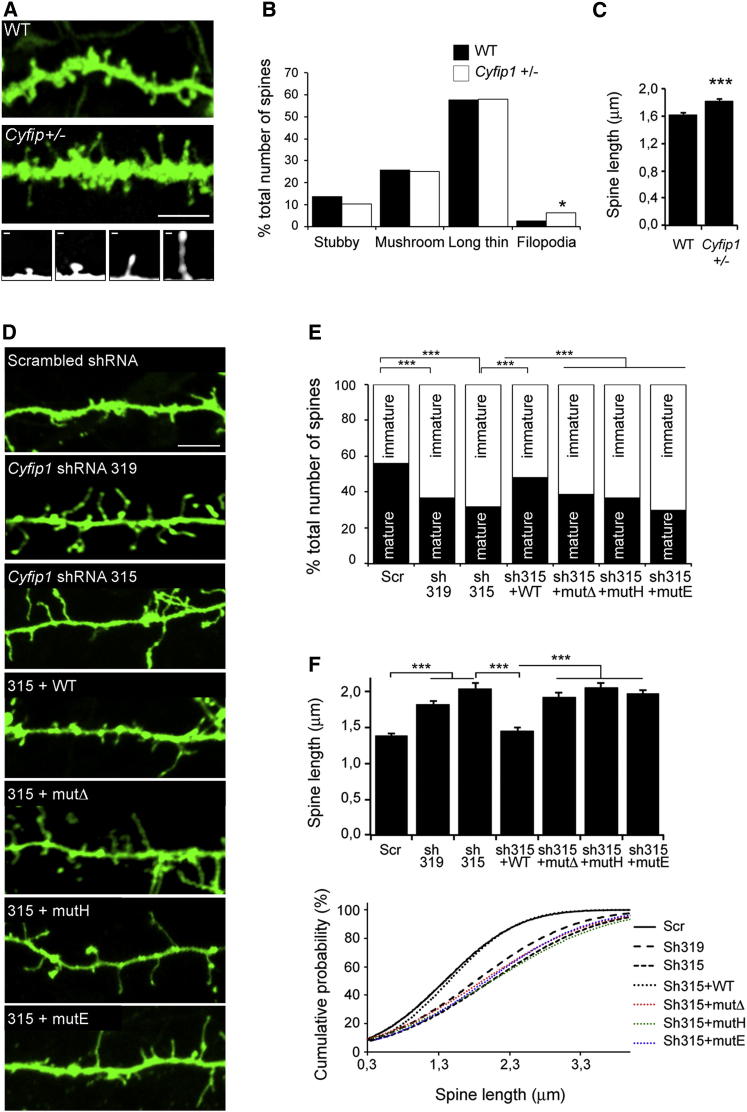
CYFIP1 Is Required for Correct Spine Morphology (A) Dendritic spines are defective in ex vivo cortical neurons from *Cyfip1*^*+/−*^ mice. Cortical neurons were labeled with DiI by diolistic staining on brain slices. Panels show representative dendritic sections; scale bar represents 5 μm. Insets in the lower panel represent magnification of individual spines classified as mature (stubby and mushroom-like) and immature (long thin and filopodia). Scale bar represents 0.5 μm. (B) Dendritic spine morphology in cortical neurons from WT (black) or *Cyfip1*^+/−^ (white) animals. Distribution of spines as percentage is shown (n = 282 WT; n = 310 *Cyfip1*^+/−^; χ^2^ test, ^∗^p < 0.05). Bars represent mean ± SEM. (C) Mean spine length in WT and *Cyfip1*^+/−^ neurons (Student’s t test, ^∗∗∗^p < 0.001). Bars represent mean ± SEM. (D) Dendritic spines are altered in cultured cortical neurons silenced for *Cyfip1*. Outline of dendritic shafts from DIV14 primary cortical neurons transfected with scrambled, two *Cyfip1* shRNAs (shRNA 319, 315), or shRNA 315 cotransfected with RNAi-resistant CYFIP1 WT, mutΔ, mutH, or mutE. Panels show representative dendritic sections; scale bar represents 5 μm. (E) Dendritic spine morphology of neurons shown in (D), expressed as percentage of mature (in black, stubby + mushroom-like) and immature (in white, long thin + filopodia) spines (at least ten neurons/condition, χ^2^ test, ^∗∗∗^p < 0.001). (F) Upper panel: mean spine length of neurons shown in (D) (one-way ANOVA with Bonferroni correction, ^∗∗∗^p < 0.001). Lower panel: Cumulative probability plots for mean spine length. Bars represent mean ± SEM. See also Figure S5.

Alterations in factors controlling protein synthesis (e.g., FMRP) or actin remodeling (e.g., WAVE1) cause dendritic spine defects (Irwin et al., 2001, Kim et al., 2006). Therefore, we addressed the question of whether CYFIP1 plays a role in dendritic spine formation by studying mice deficient in *Cyfip1*. Brain slices were isolated from *Cyfip1*^+/–^ and WT littermates, and individual neurons were labeled diolistically. Dendritic spines were measured and assigned to four morphological classes, namely mature (stubby and mushroom) and immature types (long thin and filopodia). Neurons displayed a spine distribution in agreement with previous ex vivo studies (Galvez and Greenough, 2005, Irwin et al., 2002). Neurons from *Cyfip1*^+/–^ mice, despite the mild reduction in CYFIP1, showed an increased population of filopodia (Figures 5A–5C), but no defects in spine density and head width (data not shown). To reduce CYFIP1 expression more drastically, primary cortical neurons (DIV9) were silenced for *Cyfip1* (sh319 and sh315, or scrambled shRNA), and spine density and morphology were examined at DIV14. Neuronal morphology was outlined by a farnesylated GFP (F-GFP) carried by the shRNA construct (Figures 5A–5D and S5E). Spines were classified as above. Spine number and spine measurements in cortical neurons treated with scrambled shRNA were consistent with previous reports (Papa et al., 1995). Spine density did not differ significantly between scrambled and *Cyfip1* shRNA neurons (not shown). However, *Cyfip1* knockdown robustly affected spine morphology: spines with mature phenotype (i.e., “stubby” and “mushroom”) were significantly reduced in *Cyfip1*-silenced neurons compared to control, whereas elongated, immature-looking spines increased in number (Figures 5D, 5E, S5E, and S5F). Mean head width was unchanged (not shown), but mean spine length was increased as a consequence of *Cyfip1* silencing; cumulative probability plots corroborated these results (Figure 5F). To exclude the possibility that the phenotype might be due to off-target effects, we performed a rescue experiment by cotransfecting the sh315 (against *Cyfip1* 3′UTR) and the *Cyfip1* WT coding sequence. The construct was able to restore normal CYFIP1 levels (Figure S5E), and consequently proper spine distribution (Figures 5D, 5E, and S5F) and mean spine length (Figure 5F).

Finally, we aimed at investigating the contribution of CYFIP1-eIF4E and WRC to spine formation. Therefore, we cotransfected the CYFIP1 mutants validated above with *Cyfip1* sh315 to knockdown the endogenous protein (Figure S5E), and analyzed dendritic spine morphology. All mutants failed to restore the normal spine distribution and spine length (Figures 5D, 5F, and S5F), indicating that both CYFIP1 complexes are equally important for proper spine formation.

In conclusion, CYFIP1 deficiency alters the proper functioning of two complexes modulating critical synaptic processes, i.e., protein synthesis and actin cytoskeleton remodeling, both of these ultimately leading to defects in spine morphology.

### CYFIP1 Interactome Is Linked to Human Disease

**Figure 6:**
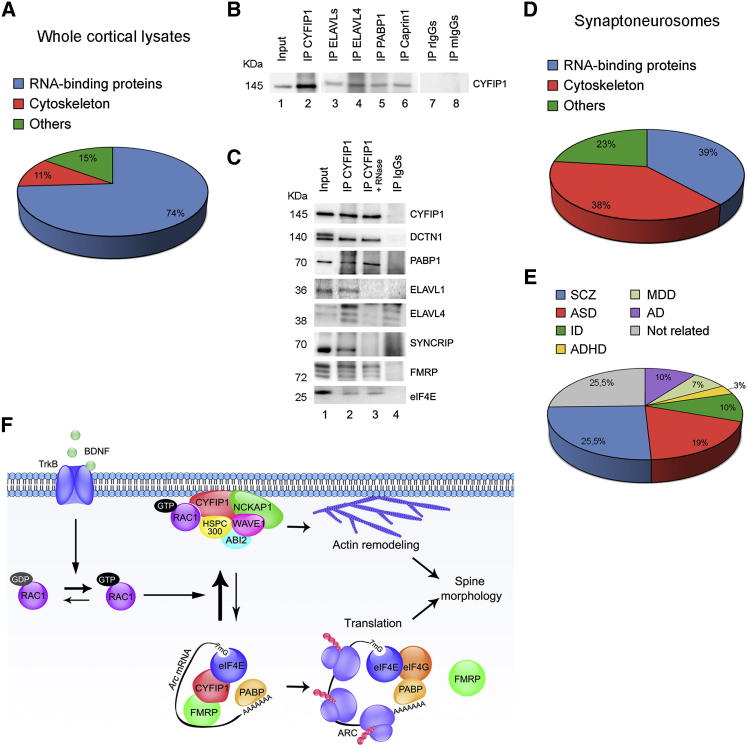
The CYFIP1 Interactome in Mouse Cortex and Synapses and Its Relevance for Neuropsychiatric Disorders (A) CYFIP1 interactome, as revealed by MS of the proteins coimmunoprecipitating with CYFIP1 in cortical whole-cell lysate. IP with rabbit IgGs was used as a negative control; n = 3. The identified proteins are listed in Table S2, Table S3. (B) Validation of CYFIP1 interactors by reverse IP. Lane 1, input (1/100); lanes 2–6, specific IPs; lanes 7–8, controls with rabbit and mouse IgGs. See also Figure S6. (C) CYFIP1 interactome is partially RNase sensitive. Lane 1, input (1/50); lane 2, CYFIP1 IP; lane 3, CYFIP1 IP after RNase treatment; lane 4, control IP (rabbit IgGs); n = 5. (D) Outcome of MS analysis of the proteins coimmunoprecipitating with CYFIP1 in cortical synaptoneurosomes. IPs with rabbit IgGs were used as negative control; n = 6. The identified proteins are listed in Table S4. (E) Many CYFIP1 interactors are linked to neurological diseases. Indicated are the percentages of genes related to intellectual disability (ID), autism spectrum disorder (ASD), attention deficit hyperactivity disorder (ADHD), schizophrenia (SCZ), major depressive disorder (MDD), and Alzheimer’s disease (AD) (the diagram is approximate, as some genes are related to more than one disease; see also Table S5). (F) Proposed model for the interplay of CYFIP1 complexes in brain. In neurons, CYFIP1 is associated with two distinct protein complexes, the CYFIP1-FMRP-eIF4E, which represses translation of specific mRNAs such as *Arc/Arg3.1* mRNA, and the WRC (CYFIP1-NCKAP1-WAVE1-ABI2-HSPC300), which regulates actin remodeling. BDNF signaling activates Rac1, and GTP-Rac1 changes the equilibrium between the two CYFIP1 complexes by inducing a conformational change that releases CYFIP1 from eIF4E and relocates it to active WRC. As a consequence, actin cytoskeleton is remodeled and, concomitantly, the translation of proteins that encode cytoskeleton elements and synaptic function and plasticity is activated. The two processes converge to regulate proper spine morphology, which is compromised by perturbations of CYFIP1 expression or interference with CYFIP1-eIF4E and CYFIP1-WRC.

To further expand the knowledge of CYFIP1 in the brain, we studied its interactome in mouse cerebral cortex through immunoprecipitation with a specific anti-CYFIP1 antibody and tandem mass spectrometry (MS). In whole cortical lysates, we identified a total of 27 CYFIP1-associated proteins, of which 74% are RNA-binding proteins (RBPs) (Figure 6A), comprising either known (FMRP and PABP-1) (Napoli et al., 2008, Schenck et al., 2003) or novel (ELAV-like proteins, Caprin1 and hnRNPQ/SYNCRIP) partners; these are listed in Tables S2 and S3. The association of some interactors (ELAVL4, PABP1, Caprin1, SYNCRIP, FMRP, eIF4E, and DCTN1) was validated by reverse immunoprecipitation (Figures 6B and S6). To investigate whether these interactions depend upon RNA, CYFIP1 was immunoprecipitated from RNase-treated cortical lysates. Whereas the binding of CYFIP1 to PABP1, DCTN1 and eIF4E was not compromised by RNA degradation, the interaction with SYNCRIP, ELAVL4, and ELAVL1 was no longer detected (Figure 6C) implying that the CYFIP1 complexes contain both protein and RNA molecules. The association of FMRP with CYFIP1 was slightly reduced by treatment with RNase, confirming previous indications that RNAs (e.g., BC1) can strengthen this interaction (Napoli et al., 2008) In conclusion, the mouse cortical CYFIP1 interactome consists mainly of proteins that regulate mRNA metabolism and translation.

Because CYFIP1 is abundant at synapses (Figure S1), we immunoprecipitated CYFIP1 from mouse cortical synaptoneurosomes. Sixteen proteins were identified by MS, seven of which had not been detected in the cortical lysate data set likely because they are enriched in the CYFIP1 complexes in the synaptic compartment. The synaptic CYFIP1 interactome contained not only RBPs, but also cytoskeleton-related proteins, including components of the WRC (NCKAP1, ABI1/2, and WAVE1; Figure 6D; Tables S3 and S4). These results further demonstrate that CYFIP1 is active in regulating mRNA translation and determining cytoskeleton-based cell morphology.

Deletions and duplications of a chromosomal region including *CYFIP1* have been linked to ID, ASD, and schizophrenia (Cooper et al., 2011, Doornbos et al., 2009, Leblond et al., 2012, Murthy et al., 2007, Nowicki et al., 2007, Tam et al., 2010, van der Zwaag et al., 2010, von der Lippe et al., 2010, Zhao et al., 2012). We reasoned that proteins in the same protein network might have a similar pathological effect. A literature search on disease involvement of the genes in question revealed that 25 out of the 40 proteins that bind CYFIP1 are encoded by genes associated with ID, ASD, ADHD, schizophrenia, major depressive disorder, and Alzheimer’s disease (Tables S4 and S5). In addition, a gene-based analysis interrogating for association with schizophrenia based on the meta-analytic p values obtained by the largest schizophrenia genome-wide association study to date (Ripke et al., 2011) (9,394 cases and 12,462 controls) revealed that 8 out of 36 tested autosomal genes of the CYFIP1 interactome had a nominally significant p value (<0.05) for association with schizophrenia (Tables S4–S6). This significantly exceeds the expectation (1.8 genes) under the null hypothesis of no association (one-sided Fisher’s exact test, p = 0.042), although the polygenic nature of schizophrenia should be considered. One gene, FAM120A, was significantly associated with schizophrenia (p = 0.00064) after Bonferroni correction for testing 36 genes. In summary, 25 proteins out of 40 identified in our CYFIP1 interactome are encoded by genes involved in diseases: 26% are associated with schizophrenia, 19% with ASD, and 10% with ID (Table S4; Figure 6E). These observations suggest that CYFIP1 and its interaction partners are linked to pathways that, if impaired, can be associated with intellectual disabilities and psychiatric disorders.

## Discussion

CYFIP1 is present in two functional complexes essential for synaptic morphology and function: a ribonucleoparticle repressing protein synthesis and the WAVE regulatory complex (Figure 6F). When CYFIP1 interacts with NCKAP1 forming a platform for the assembly of the WRC, the interaction with eIF4E is obstructed and vice versa. The segregation into the two complexes relies on alternative CYFIP1 conformations, a planar one needed for the WRC (Chen et al., 2010), and a more globular one for the interaction with eIF4E. BDNF, a neurotrophin and synaptic plasticity-inducing factor, able to induce protein synthesis (Takei et al., 2004) and cytoskeleton rearrangements (Bramham, 2008), reduces the pool of CYFIP1 repressing translation and concomitantly increases the amount of CYFIP1 recruited on the WRC. This event is regulated by Rac1 and is facilitated by a conformational change, as shown by FRET experiments: after BDNF administration, CYFIP1 switches from a more globular form to a planar conformation suitable for incorporation in the WRC. As a consequence, CYFIP1 is freed from eIF4E and the synthesis of key modulators of synaptic plasticity such as ARC is activated (Figure 6F). Enhanced expression of ARC, in the absence of CYFIP1 or FMRP, might alter AMPA receptor endocytosis and affect the actin cytoskeleton, therefore affecting synaptic structure and physiology (Shepherd and Bear, 2011). Concomitant to ARC induction, active Rac1 promotes CYFIP1 recruitment to the WRC and thus actin polymerization. In line with our evidence, Rac1 activation was shown to translocate CYFIP1 to actin-rich domains involved in cellular protrusions in mouse fibroblasts (Castets et al., 2005). Also, CYFIP1 overexpression in *Drosophila* rescues eye defects caused by a constitutively active Rac1 mutant (Schenck et al., 2003); in light of our results, this overexpression might improve the balance in CYFIP1 partitioning between the two complexes caused by the increased Rac1 signaling.

Dendritic spine maturation is critical for correct brain functioning (Penzes et al., 2011). We show here that CYFIP1 depletion severely affects dendritic spine morphology both in vivo and in vitro, causing an unbalanced ratio between mature and immature spines (Figures 4 and 5). Downregulation of *Cyfip1* causes defects in ARC synthesis and actin polymerization in dendritic spines (Figures 3 and 4). Altering CYFIP1 incorporation in the WRC (as with mutant H) affects F-actin polymerization but not ARC synthesis; conversely, when the CYFIP1-eIF4E interaction is impaired (as with mutant E), ARC synthesis is altered with no effect on F-actin levels (Figure 4). Our studies reveal that correct spine morphology requires both intact CYFIP1-eIF4E and CYFIP1-WRC complexes, and that correct coordination between the two is essential for proper ARC synthesis, actin polymerization, and finally spine morphology (Figures 5 and 6).

Effects of CYFIP1 reduction on dendritic spines are compatible with the enhanced mGluR-dependent LTD and behavioral abnormalities caused by *Cyfip1* haploinsufficiency (Bozdagi et al., 2012), similar to the phenotype observed in *Fmr1* KO mice (Bear et al., 2004). ARC is required for mGluR-LTD and AMPAR internalization (Waung et al., 2008), and we show that *Cyfip1*^+/−^ mice have excessive ARC at synapses (Figure 3D). Furthermore, the observed spine dysmorphogenesis is in line with defects in the WRC components (Grove et al., 2004, Kim et al., 2006, Soderling et al., 2007), loss of FMRP (Comery et al., 1997, Cruz-Martín et al., 2010, Galvez and Greenough, 2005, Irwin et al., 2002), overexpression of ARC (Peebles et al., 2010), or Rac1 blockade during early development (Tashiro and Yuste, 2004). All these phenotypes overlap, without being identical, indicating that CYFIP1 is at the hub of more than one spine-controlling pathway.

Spine dysmorphogenesis is a common feature of several neuropsychiatric disorders (Penzes et al., 2011). Of note, FMRP- and CYFIP1-linked disorders are characterized by spine dysmorphogenesis that we show here is caused by an imbalance of protein synthesis and actin remodeling. *CYFIP1* is implicated in ID (Cooper et al., 2011, Napoli et al., 2008, Nowicki et al., 2007, Schenck et al., 2003), ASD (Cooper et al., 2011, Doornbos et al., 2009, Murthy et al., 2007, Nishimura et al., 2007, Sahoo et al., 2006, van der Zwaag et al., 2010, von der Lippe et al., 2010), and SCZ (Tam et al., 2010, Zhao et al., 2012). Consistently with the idea that related human disorders might share genetic causes because they are due to perturbations of highly interconnected cellular networks (Vidal et al., 2011), we find that the CYFIP1 interactome is enriched in genes implicated in ID, ASD, SCZ, ADHD, MDD, and AD. Importantly, the two key CYFIP1 interactors examined here, NCKAP1 and eIF4E, have been shown to be genetically associated with ASD (Iossifov et al., 2012, Neves-Pereira et al., 2009). Our findings suggest that mutations in the CYFIP1 network might explain part of the autistic features observed in FXS patients (Farzin et al., 2006), which can also suffer from psychosis (Reiss et al., 1986).

Mutations in the genes of the CYFIP1 interactome might perturb the homeostasis of the interaction networks, regulating translation versus cytoskeleton remodeling, thereby triggering a spectrum of pathological processes at synapses that can lead to a broad range of clinical manifestations, such as intellectual disabilities, autism, and schizophrenia.

## Experimental Procedures

### Animal Care, Strain, and Stage

Animal care was conducted according to the Belgian law of August 14th, 1986, concerning the protection and well-being of animals, and the following Koninklijk Besluit (K.B.) of November 14th, 1993 and K.B of September 13th, 2004, as well as to the European Community Council Directive 86/609, Oja L 358, 1, December 12, 1987, and international guidelines (European Community Council Directive 86/609, Oja L 358, 1, December 12, 1987; National Institutes of Health Guide for the Care and Use of Laboratory Animals, US National Research Council, 1996). One-month-old C57BL/6J *Fmr1* KO and WT control littermates were used for the EM-IHC control.

WT mice used in this study were 3- to 4-week-old males C57BL/6J. Two-month-old *Cyfip1*^+/−^ 129/Sv C57BL/6J and WT control littermates were used for diolistic staining on brain slices.

### Immunoprecipitation

Immunoprecipitation (IP) was performed as previously described (Napoli et al., 2008). For details see the Supplemental Experimental Procedures.

### Neuronal Transfection

Mouse primary cortical neurons (DIV9) were transfected with plasmids carrying scrambled or *Cyfip1* shRNA and *Cyfip1* WT or mutants using a calcium phosphate method (Sans et al., 2003). At DIV14, neurons were fixed for 20 min in PFA/SEM (4% PFA, 0.12 M sucrose, 3 mM EGTA, 2 mM MgCl_2_ in PBS).

### Immunofluorescence

Primary cortical neurons were fixed with 4% paraformaldehyde (PFA/SEM), permeabilized with 0.2% Triton X-100, and incubated overnight with the antibodies, as indicated in the Supplemental Experimental Procedures. Confocal images were obtained as described in the Supplemental Experimental Procedures.

### Immunoblotting

Immunoblotting was performed using standard protocols. Antibodies list and usage is described in the Supplemental Experimental Procedures.

### Diolistic Staining of Ex Vivo Brain Slices

See the Supplemental Experimental Procedures.

### Imaging

A confocal laser-scanning microscope (Nikon) with 40× or 60× oil objectives with sequential acquisition setting at 2,048 × 2,048 pixels resolution was used. For immunofluorescence (IF), only a z series was acquired; for spine analysis, each image was a z series projection, of ∼7 to 9 images each, averaged two times and taken at 0.8 μm depth intervals.

### Dendritic Spines Analysis

Images were processed and analyzed with ImageJ 1.44 software. Five 20 μm segments starting at least 25 μm from the cell soma were analyzed for each neuron. F-EGFP or DiI staining was used to outline the profile of the dendritic shaft and protrusions. Maximal spine head width (W_H_), neck width (W_N_), and length (L) were measured for each dendritic protrusion. Spines were defined as follows: Stubby (L ≤ 1 μm), Mushroom (1 < L ≤ 3 μm; W_H_ ≥ 2 × W_N_), Long Thin (1 < L ≤ 3 μm; W_H_ < 2 × W_N_), and Filopodia (3 < L ≤ 5 μm). At least ten randomly chosen neurons/condition from three independent cultures were imaged for quantification. Counts and data analysis were conducted blind to experimental condition.

### Synaptoneurosomes Purification and Stimulation

Cortical synaptoneurosomes were prepared as previously described (Napoli et al., 2008). Pre- and postsynaptic fractions were isolated from cortical synaptoneurosomes as previously described (Phillips et al., 2001). See the Supplemental Experimental Procedures for details.

### Neuronal Cell Cultures and BDNF Stimulation

Primary mouse cortical neurons were prepared as previously described (Ferrari et al., 2007). See the Supplemental Experimental Procedures for details and treatments with BDNF, cycloheximide, and actinomycin D (Sigma).

### m^7^GTP Chromatography

The procedure was slightly modified from Napoli et al. (2008). See the Supplemental Experimental Procedures for details.

### DNA Constructs

See the Supplemental Experimental Procedures.

### Lentiviral Production to Silence *Cyfip1*

HEK293T cells were used as packaging cells and transfected by the calcium phosphate method (Chen and Okayama, 1987) with second generation plasmids (pLKO.1, Mission shRNA, Sigma-Aldrich) (Naldini et al., 1996) carrying scrambled or *Cyfip1* shRNAs. See the Supplemental Experimental Procedures for details.

### FRET/FLIM Experiment in Primary Neurons

Mouse primary cortical neurons (DIV 9) were transfected with the indicated DNA constructs using Lipofectamine 2000. Neurons were treated with 100 nM of the panTrk inhibitor K252a for 24 hr or 100 ng/ml BDNF for 30 min. Coverslips were then fixed with 4% PFA for 20 min, washed with PBS, incubated with 1 M NH_4_Cl for 15 min, washed, and then mounted with Mowiol. A construct carrying a tandem mCherry-EGFP was used as positive control for intramolecular FRET. Two constructs carrying mCherry and EGFP (Clontech) separately were cotransfected to provide a negative control. FRET/FLIM measurements were performed as in Zhang et al. (2013). For details see the Supplemental Experimental Procedures.

### Computational Studies

See the Supplemental Experimental Procedures.

### Disease Annotation and Gene-Based Analyses

See the Supplemental Experimental Procedures.

### Statistics

Comparisons between two groups were performed using one-sample or two-sample two-tailed Student’s t test. One-way or two-way ANOVA followed by post hoc Student’s t test with Holm’s or Bonferroni correction were used for multiple comparisons. Distributions were analyzed using Pearson’s χ^2^ test. Comparisons between cumulative probability plots were performed using two-sample Kolgomorov-Smirnov (K-S) test. Significance was accepted to p < 0.05. Bars represent SEM.

## Supplemental Information

Document S1. Supplemental Experimental Procedures and Figures S1–S6

Document S2. Tables S1–S6 Table S1. Surface Accessible Surface (SAS) of the Lys743, (side chain and backbone) for the free CYFIP1 or CYFIP1 embedded within the WRC. Table S2. MS analysis of the proteins co-immunoprecipitating with CYFIP1 in whole cortical lysates. Swissprot protein names and accession numbers are reported. The number of single peptides is also indicated. Table S3. Aliases of the CYFIP1 co-interacting proteins identified by MS. Swissprot name, Swissprot and MGI accession number, and a list of gene/protein aliases are reported. Proteins are reported in alphabetical order. Table S4. CYFIP1 interactome at synapses. MS analysis of the proteins co-immunoprecipitating with CYFIP1 in cortical synaptoneurosomes. Protein names and accession numbers are reported according to Swissprot. The number of single peptides is also indicated. Table S5. Involvement of the CYFIP1 interactors in neuropsychiatric disorders. Swissprot name, Gene ID, OMIM ID of CYFIP1 interactors is reported. The genes were annotated for their involvement in diseases based on literature or GWAS study (Schizophrenia). The following disorders were annotated: Intellectual Disability (ID), Autism Spectrum Disorder (ASD), Attention Deficit Hyperactivity Disorder (ADHD), Schizophrenia (SCZ), Major Depressive Disorder (MDD) and Alzheimer's disease (AD). The last column indicates the p value of the association of the gene with Schizophrenia using VEGAS software. Table S6. Gene-based association results for CYFIP1 co-interactors in Schizophrenia. Gene names of the CYFIP1 co-interactors are reported together with the total number of SNPs available in the gene, and the gene-based p-value and the name of the most significant SNP.
