# Supplementary material for: CYFIP1 Coordinates mRNA Translation and Cytoskeleton Remodeling to Ensure Proper Dendritic Spine Formation
Source: Neuron. 2013 Sep 18;79(6):1169–82. doi: 10.1016/j.neuron.2013.06.039 (PMC3781321; doi:10.1016/j.neuron.2013.06.039)
Supplement: Document S1. Supplemental Experimental Procedures and Figures S1–S6 [file mmc1.pdf]

Neuron, Volume *79*

Supplemental Information

CYFIP1 Coordinates mRNA Translation and Cytoskeleton Remodeling to Ensure Proper Dendritic Spine Formation

Silvia De Rubeis, Emanuela Pasciuto, Ka Wan Li, Esperanza Fernández, Daniele Di Marino, Andrea Buzzi, Linnaea E. Ostroff, Eric Klann, Fried Zwartkruis, Noburo H. Komiyama, Seth Grant, Christel Poujol, Daniel Choquet, Tilmann Achsel, Danielle Posthuma, August B. Smit, and Claudia Bagni

Figure Legends

Supplementary Figure 1. CYFIP1 and FMRP presence in dendrites and spines. (A) Electron micrographs of DAB-immunohistochemistry for CYFIP1 and FMRP on the *stratum radiatum* of the hippocampus. Arrowheads show stained dendrites, spines or axons; scale bar, 1 μm. **(B)** Histogram of the distribution of DAB-positive items (Axons, white; Dendrites, grey; Spines, black) representing the percentage of each group over the total number of counts (413 counts for CYFIP1, 725 for FMRP). Bars represent mean ± SEM. **(C-F)** EM controls using: **(C)** the anti-CYFIP1 antibodies upon pre-adsorption with the peptide used to generate the antibody; **(D)** the anti-FMRP antibody upon pre-absorption with the FMRP protein domain used to generate the antibody; **(E)** omission of primary antibodies; **(F)** *Fmr1* KO animals. Scale bar, 1 μm. **(G)** Distribution of CYFIP1, FMRP and eIF4E in biochemically fractionated synaptoneurosomes. Upper panel, immunoblot analysis to detect CYFIP1, FMRP and eIF4E on synaptoneurosomal fractions. Synaptophysin, Syntaxin, NR2B, GluR4 and PSD-95 were used as markers of the three fractions (Phillips et al., 2001). Lane 1, total cortex extracts; lane 2, cortical synaptoneurosomes; lane 3, synaptic soluble fraction; lane 4, insoluble pre-synapses; lane 5, insoluble post-synapses. Lower panel, histogram representing the distribution of the proteins in the three fractions. Bars represent mean ± SEM.

Supplementary Figure 2. CYFIP1, eIF4E and NCKAP1 expression and distribution in neurons. (A) CYFIP1, FMRP and eIF4E expression profile in primary cortical cultures at different stages. Days in vitro (DIV) 1, 2, 7, 14, 21 and 28. Coomassie staining was used for normalization. **(B)** Images represent the single stainings of the enlargements (white boxes) shown in Figure 2A. CYFIP1 is shown in green, eIF4E in red and NCKAP1 in blue. Scale bar, 5 μm. **(C)** Co-localization of CYFIP1 with NCKAP1 and eIF4E after BDNF stimulation at 0, 5, 15, 30, 60 min. One-way ANOVA with Holm's *post hoc* test, *$p< 0.05$; *** $p< 0.001$. n > 20. Bars represent mean ± SEM. **(D)** Mouse cortical extracts were prepared with and without detergent and analyzed on a BN-PAGE. Lanes belong to the same blot and were incubated with different antibodies as indicated:

lane 1, anti-CYFIP1; lane 2, anti-NCKAP1; lane 3, anti-eIF4E), *n*=4. **(E)** NSC23766 is effective in blocking BDNF-induced Rac1 activation. Primary neurons were stimulated with BDNF +/- NSC23766 and active Rac1 was measured using a PAK1 PBD affinity chromatography (*n* = 3, Student's *t* test, * p < 0.05). Bars represent mean ± SEM.

Supplementary Figure 3. Dynamics of EYFP-CYFIP1, Cerulean-NCKAP1 and eIF4E-mCherry after BDNF stimulation. Neurons were transfected with pEYFP-CYFIP1 (green), pCerulean-NCKAP1 (blue) and eIF4E-pmCherry (red), and time-lapse images were taken every 10 sec for 20 min starting 10 min after BDNF (100 ng/ml) stimulation. **(A)** Z projection of the pEYFP-CYFIP1 channel of a representative neuron, the white arrow points to the spine shown in (B). Scale bar, 10 µm. **(B)** For the spine highlighted in (A), one image for every minute is shown with all three channels. White circles outline CYFIP1 complexes defined as areas of pEYFP-CYFIP1 enrichment. Fluorescence intensity for the three proteins was measured inside these regions. Scale bar, 1 µm. The plot in **(C)** reports the relative fluorescence intensity values of Cerulean-NCKAP1 and eIF4E-mCherry normalized to pEYFP-CYFIP1. RSQ = square of the correlation coefficient.

Supplementary Figure 4. CYFIP1-eIF4E complex dynamics upon translation-inducing stimuli in serum-starved cells and effects of the Trk and Rac1 inhibitors (A) Bright fields images of serum-starved HEK293T cells. Cells were serum-deprived for 17 hours and the medium was replenished with serum for 5, 10 or 60 min. The images show the morphological changes caused by serum starvation. **(B)** Upper panel, m^7GTP chromatography on HEK293T cells. Lanes 1-4, input (1/100) at 0, 5, 10 or 60 min after serum restoration; lanes 4-8, pull down at 0, 5, 10 or 60 min. Lower panel, CYFIP1 and FMRP signals were normalized to eIF4E and plotted against time after serum addition (*n* = 3, Student's t test, * *p* < 0.05). Bars represent mean ± SEM. **(C)** Left panel, serum-starved HEK293T cells were stimulated with serum to activate translation with and without Rac1 inhibition by NSC23766. Lanes 1-2, input (1/100) of vehicle-treated cells at 0 and 5 min after serum restoration; lanes 3-4, m^7GTP pull down on vehicle-treated cells at 0 and 5 min after serum addition; lanes 5-8, as lanes 1-4 with NSC23766-treated cells. Right panel, CYFIP1 and FMRP signals were normalized to eIF4E and their association with eIF4E after 5 min expressed as percentage to the control (t = 0 min) in cells treated with vehicle (black) or NSC23766 (white) (*n* = 6, Student's *t* test, * p < 0.05, ** p < 0.01). Bars represent mean ± SEM. **(D)** CYFIP1 exists in a globular conformation in HEK293 cells. FRET is measured as reduction in fluorescence lifetime. Top to bottom: free mCherry and EGFP (negative control); tandem mCherry-EGFP (intramolecular FRET positive control); mCherry–CYFIP1–EGFP in serum starved cells (17 hrs); mCherry–CYFIP1–EGFP in cells treated with NSC23766; *n* = 30, one-way ANOVA with Bonferroni's *post hoc* test, ** p < 0.01, *** p < 0.001. Bars represent mean ± SEM. **(E)** Effects of panTrk inhibitor on neuronal protein synthesis. Immunoblot on neurons treated with vehicle (lane 1), panTrk inhibitor

k252a (lane 2) or BDNF (lane 3). ARC, eIF4E, phospho-eIF4E and GAPDH proteins were analyzed. **(F)** The effects of Rac1 inhibitor are not due to interference with the TrkB signalling. Immunoblot analysis for the phosphorylation of TrkB and the downstream effector ERK1/2 in cortical neurons stimulated with BDNF. BDNF robustly activates pTrkB and pERK1/2 and NSC23766 does not alter this effect. A Dynamin inhibitor (Dynasore) was used to reduce TrkB signalling.

Supplementary Figure 5. Effects of WT and mutant CYFIP1 in primary neurons (A) Silencing of *Cyfip1* in primary neurons. Left panel, immunoblot analysis of primary cortical neurons (lanes 1-3) infected with viruses transducing a scrambled shRNA plasmid (lanes 4-6) or shRNA plasmids targeting *Cyfip1*, i.e. shRNA 315 (lanes 7-9), shRNA 318 (lanes 10-12), and shRNA 319 (lanes 13-15). CYFIP1 and GAPDH immunosignals are shown. Right panel, quantification of CYFIP1 levels normalized to GAPDH and represented as percentage to not infected control (n = 3, Student's t test, *** $p < 0.001$, ** $p < 0.01$). Bars represent mean ± SEM. **(B)** Regions of proposed CYFIP1–NCKAP1 interactions. CYFIP1 is shown red, NCKAP1 green and the second hydrophobic patch in cyan. **(C)** Expression of the CYFIP1 constructs in HEK293T cells for 24 and 72 hrs. Asterisks indicate exogenous EYFP-tagged proteins. **(D)** Validation of the siRNA used to silence human *CYFIP1* in HEK293T cells. 20 or 60 pmol of scrambled or *CYFIP1* siRNAs (1+2) were tested, and 60 pmol were used for subsequent experiments. **(E)** Representative images of dendritic sections of cortical neurons transfected with scrambled shRNA, shRNA against *Cyfip1* (315) and shRNA 315 co-transfected with RNAi-resistant CYFIP1 WT, mutΔ, mutH or mutE. Farnesylated-GFP (green) outlines the dendrites, CYFIP1 staining is shown in red. **(F)** Detailed morphological distribution of dendritic spines in neurons shown in Figure 5D. White, filopodia; grey, long thin; dark grey, mushroom; black, stubby (at least 10 neurons/condition, χ^2 test, *** $p < 0.001$).

Supplementary Figure 6. Validation of CYFIP1 co-interacting partners. Part of the identified CYFIP1 interactome (Figure 6A, Table 6) was validated by reverse IPs. Lane 1, input (1/100); lanes 2–6, specific IPs; lanes 7–8, controls with rabbit and mouse IgGs.

Supplementary Table 1. Surface Accessible Surface (SAS) of the Lys743, (side chain and backbone) for the free CYFIP1 or CYFIP1 embedded within the WRC. Available separately online.

Supplementary Table 2. MS analysis of the proteins co-immunoprecipitating with CYFIP1 in whole cortical lysates. Swissprot protein names and accession numbers are reported. The number of single peptides is also indicated. Available separately online.

Supplementary Table 3. Aliases of the CYFIP1 co-interacting proteins identified by MS. Swissprot name, Swissprot and MGI accession number, and a list of gene/protein aliases are reported. Proteins are reported in alphabetical order. Available separately online.

Supplementary Table 4. CYFIP1 interactome at synapses. MS analysis of the proteins co-immunoprecipitating with CYFIP1 in cortical synaptoneurosomes. Protein names and accession numbers are reported according to Swissprot. The number of single peptides is also indicated. Available separately online.

Supplementary Table 5. Involvement of the CYFIP1 interactors in neuropsychiatric disorders. Swissprot name, Gene ID, OMIM ID of CYFIP1 interactors is reported. The genes were annotated for their involvement in diseases based on literature or GWAS study (Schizophrenia). The following disorders were annotated: Intellectual Disability (ID), Autism Spectrum Disorder (ASD), Attention Deficit Hyperactivity Disorder (ADHD), Schizophrenia (SCZ), Major Depressive Disorder (MDD) and Alzheimer’s disease (AD). The last column indicates the p value of the association of the gene with Schizophrenia using VEGAS software. Available separately online.

Supplementary Table 6. Gene-based association results for CYFIP1 co-interactors in Schizophrenia. Gene names of the CYFIP1 co-interactors are reported together with the total number of SNPs available in the gene, and the gene-based p-value and the name of the most significant SNP. Available separately online.

Supplementary Figure 1

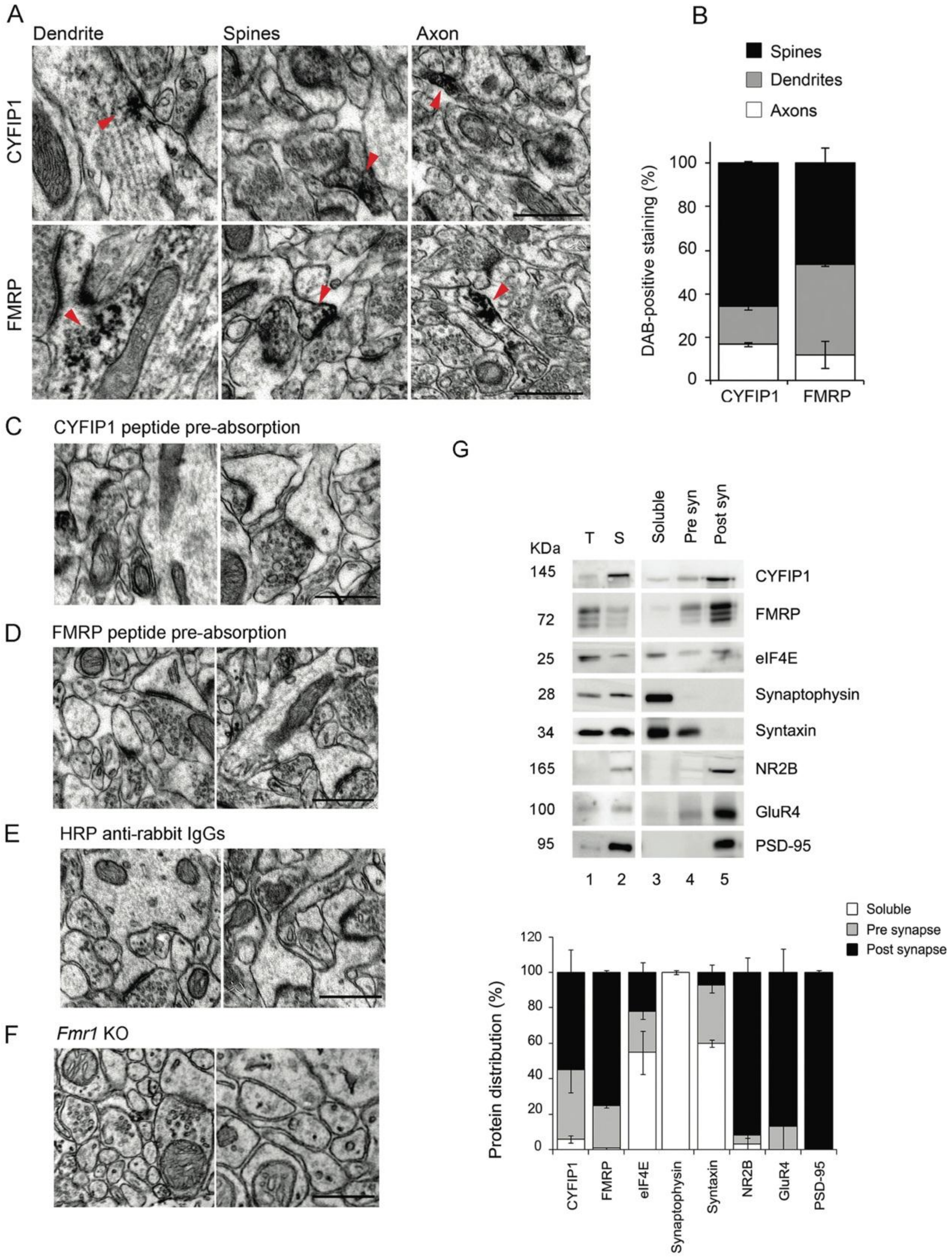

Supplementary Figure 2

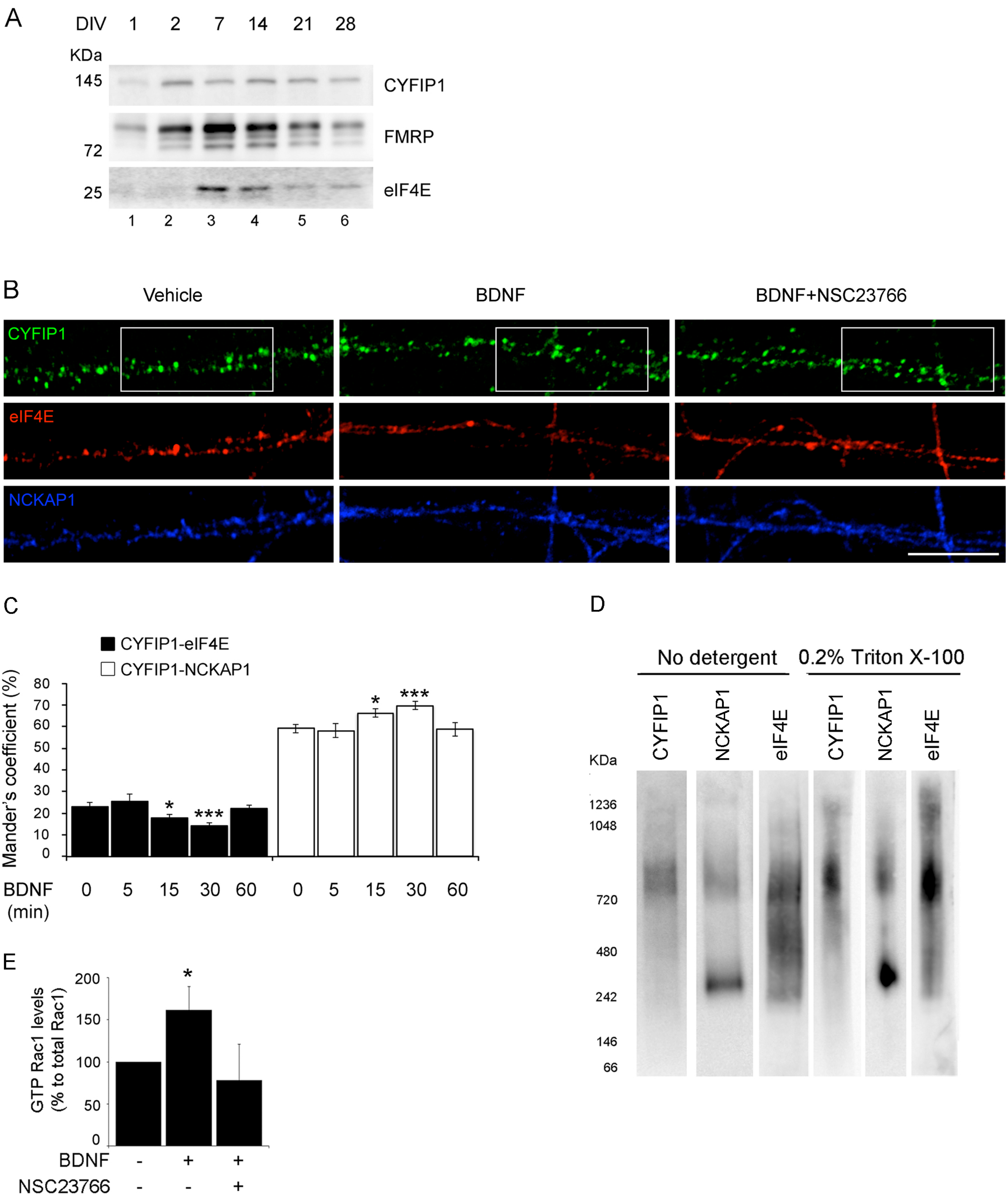

Supplementary Figure 3

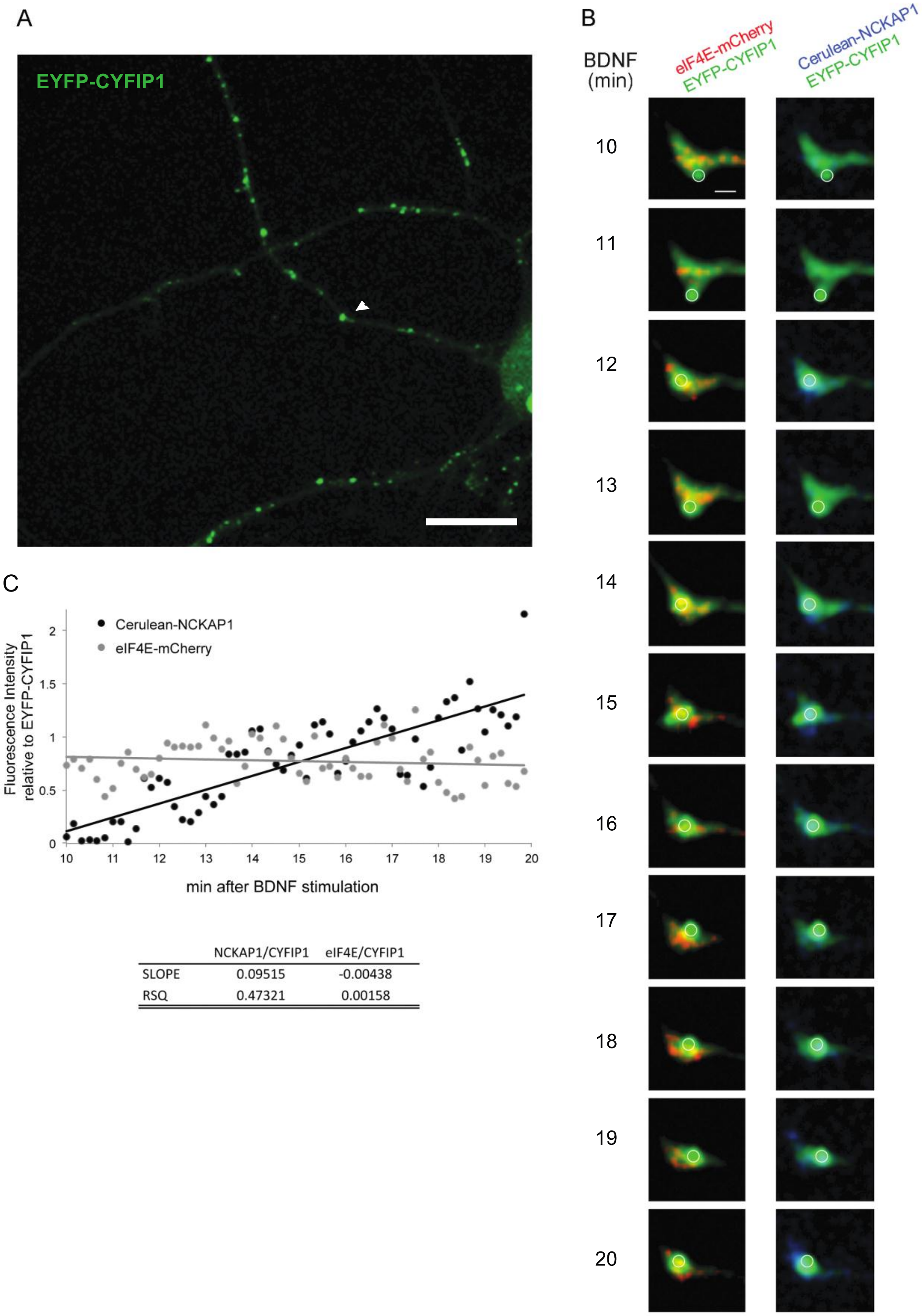

	NCKAP1/CYFIP1	eIF4E/CYFIP1
SLOPE	0.09515	-0.00438
RSQ	0.47321	0.00158

Supplementary Figure 4

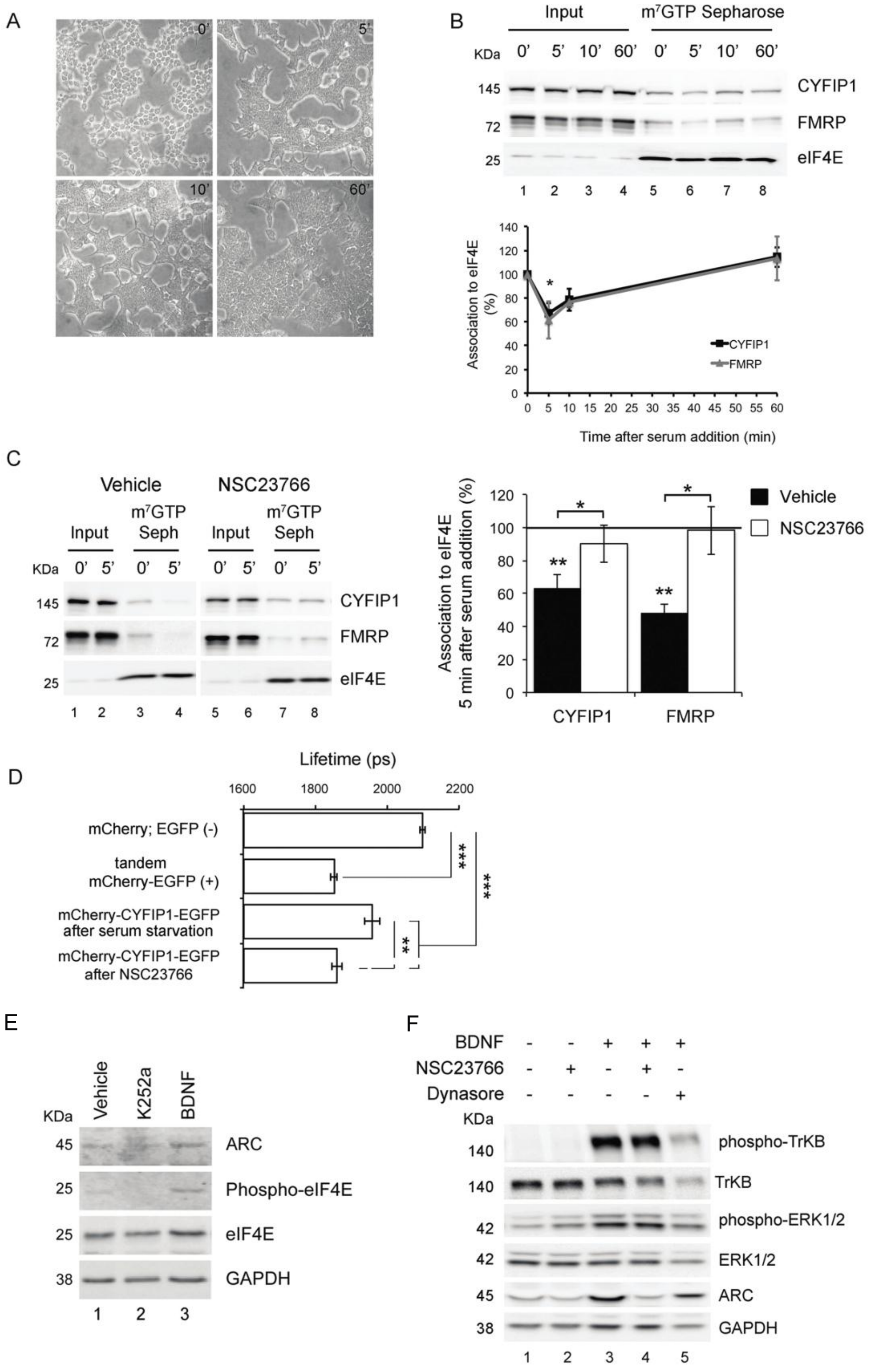

Supplementary Figure 5

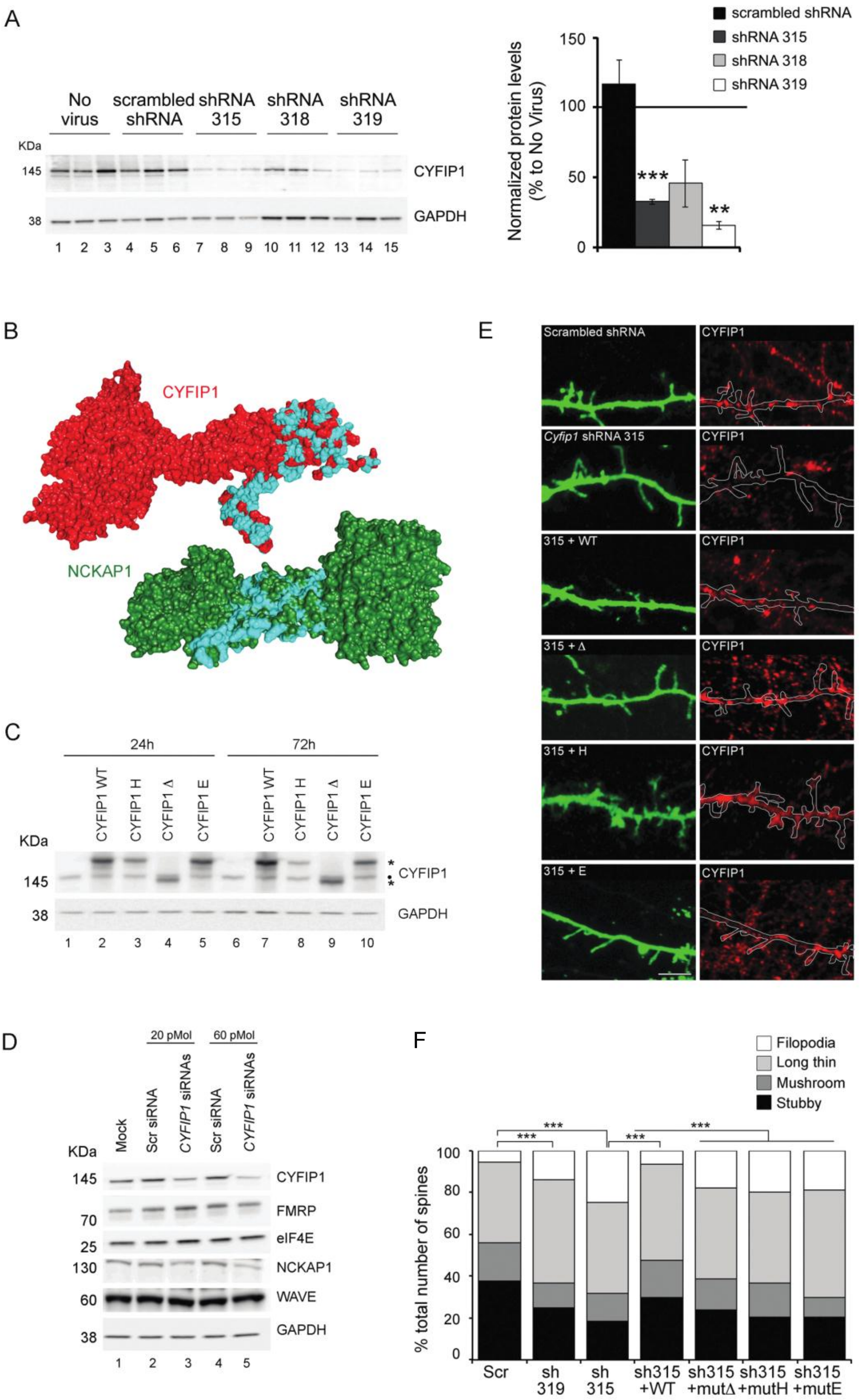

Supplementary Figure 6

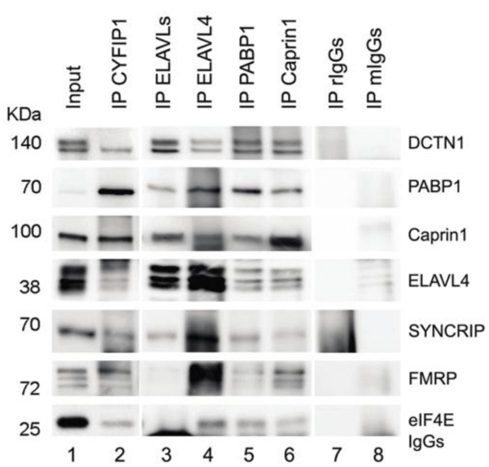

Supplemental Experimental Procedures

Liquid chromatography tandem mass spectrometry (LC-MS/MS). Samples were run on a 10% SDS-PAGE and stained with colloidal Coomassie blue. Each gel lane was cut into five slices. After destaining, proteins in the gel slice were digested with trypsin. The extracted tryptic peptides were dried in a speedvac, redissolved in 15 µL 0.1% acetic acid, and 10 µL loaded into the Eksigent nano-liquid chromatography system. The peptides were separated with a capillary reversed phase C18 column at a flow of 400 nl/min; the concentration of acetonitrile was increased linearly from 5 to 80% in 40 min, the eluted peptides were electrosprayed into the LTQ/Orbitrap MS (Thermo Scientific). The Orbitrap was operated in the range of m/z 350-2000 at a resolution of 30,000. The three most abundant precursor ions were selected for fragmentation by collision-induced dissociation with an isolation width of 2 Da. MS/MS spectra were searched against an IPI mouse database using ProteinPilot from AB-Sciex. Proteins were selected for analysis according to two criteria: (1) no detection in IP with IgGs (control) and (2) detection in at least 2 IP experiments.

Isolation of pre- and post-synaptic fractions. Pre- and post-synaptic fractions were isolated from cortical synaptoneurosomes as previously described (Phillips et al., 2001). Briefly, synaptoneurosomes were diluted in ice-cold 0.1 mM $CaCl_2$, treated for 30 min on ice with 20 mM TrisHCl, 1% Triton X-100, pH=6, and then spun to obtain the soluble fraction. The pellet was further treated with 20 mM TrisHCl, 1% Triton X-100, pH=8 and centrifuged to obtain the pre and the post-synaptic fractions. The proteins were detected by immuno-blotting and the signals quantified and normalized for the total amount of proteins stained with Coomassie Brilliant Blue.

Immuno-blotting. Immuno-blotting was performed using standard protocols. The following antibodies were used: anti-CYFIP1 (rabbit polyclonal, 1:1000) (Napoli et al., 2008), anti-FMRP (rabbit polyclonal rAMII, 1:1000) (Ferrari et al., 2007), anti-eIF4E (mouse monoclonal, 1:1000, BD Biosciences), anti-ELAVL1 (mouse monoclonal, 1:1000, Santa Cruz Biotechnology), anti-ELAVL4 (rabbit polyclonal, 1:1000, Santa Cruz Biotechnology) anti-PABP1 (rabbit polyclonal, 1:1000) (Mohr and Richter, 2003), anti-DCTN1 (goat polyclonal, 1:1000, AbCam), anti-SYNCRIP (Joachim Kremerskothen, Münster University, Germany), anti-Caprin1 (rabbit polyclonal, Proteintech), anti-Synaptophysin (mouse monoclonal, 1:1000, AbCam), anti-PSD-95 (mouse monoclonal, 1:1000, BD), anti-Syntaxin 1A (mouse monoclonal, 1:10000, Synaptic System), anti-NR2B (rabbit monoclonal, 1:1000, Upstate), anti-GluR2 (rabbit polyclonal, 1:1000, Millipore), anti-Rac1 (mouse monoclonal,1:2000, BD), anti-NCKAP1 (rabbit polyclonal, 1:1000, Novus Biologicals) anti-WAVE1 (rabbit polyclonal, 1:1000, Millipore), anti-ARC (rabbit polyclonal, 1:1000, Synaptic System), anti-GAPDH (mouse monoclonal, 1:10000, Millipore), and anti-eIF4E-P (rabbit polyclonal, 1:500, Cell signaling). HRP-conjugated anti-rabbit, anti-mouse or anti-goat antibodies (1:20000) were purchased from Promega or Millipore. Immuno-blots were developed with the chemiluminescence

technique (ECL Standard, Plus or Advance, Amersham Pharmacia) using a Fujifilm LAS-3000. Quantification was performed with AIDA software (Raytest Isotopenmeßgeräte GmbH).

Electron microscopy (EM) and immunohistochemistry (IHC). Two mice (21 days postnatal) were deeply anaesthetized with chloral hydrate and perfused transcardially with 0.5% glutaraldehyde and 4% freshly dissolved paraformaldehyde in PIPES buffer (pH = 7.4) containing 2mM $CaCl_2$, 4mM $MgCl_2$, , and. Brains were post-fixed for 1 hour, and rinsed with buffered saline (0.01M PIPES buffer, 0.9% NaCl). Brains were sectioned at 40 µm using a vibrating slicer (Leica) and sections containing the dorsal hippocampus were treated with sodium borohydride (1%, in 0.9% buffered saline, Sigma) for 30 min. Sections were rinsed in buffered saline and blocked in BSA (1%, in buffered saline, Sigma) for 90 min before incubation with primary antibodies. Sections were then incubated for 12 h with anti-CYFIP1 rabbit polyclonal (1:200)(Napoli et al., 2008) or anti-FMRP rabbit polyclonal (1:200, rAMII) (Ferrari et al., 2007) in 1% BSA. For pre-adsorption controls, primary antibodies were incubated for 1 h in a 10:1 excess of the peptide they were raised against before incubation with tissue sections. These were aa 869-886 of mouse CYFIP1 (FSQEFQRDKQPNAQPQYLC, 21st Century, USA (Napoli et al., 2008)) in DMSO (final DMSO concentration of 0.005%) for the CYFIP1 antibody, and the C-terminal domain of human FMRP expressed in E. coli (aa 516-632 (Ferrari et al., 2007)) for rAMII. Control tissue with the primary antibody omitted was also run in parallel. The rAMII antibody was also tested on tissue from an FMR1 knockout. After rinsing, sections incubated for 40 min with biotinylated goat anti-rabbit (1:200, Vector) in buffered saline with 1% BSA , rinsed,and incubated for 30 min with the avidin-biotin peroxidase complex (Vectastain Elite ABC kit, Vector). Sections were then rinsed and the peroxidase was developed with 0.025% 3,3'-diaminobenzidine tetrahydrochloride (DAB) and 0.003% hydrogen peroxide in buffered saline for 6 min.

For electron microscopy, a small area containing the stratum radiatum of the hippocampus was dissected from the sections. The tissue was transferred to 0.1M PIPES buffer and post-fixed for 1 h in reduced osmium (1% osmium tetroxide and 1.5% potassium ferrocyanide in 0.1M PIPES buffer) followed by buffer rinses and 1 h in osmium tetroxide (1% in 0.1M PIPES buffer). Tissue was rinsed in water before being dehydrated in an ascending series of ethanol dilutions, all of which contained 1.5% uranyl acetate. After rinsing in pure ethanol, the tissue was transferred to acetone and infiltrated with LX-112 resin (Ladd Chemical), flat embedded, and cured at 60°C for 48 h (Ostroff et al., 2010). Non-consecutive 45 nm-thick sections were cut on an ultramicrotome (RMC) and collected on pioloform-coated slot grids. Grids were counterstained with saturated aqueous uranyl acetate, but no lead staining was used to avoid obscuring the DAB label. Random, non-overlapping fields of the Stratum radiatum of the hippocampus were photographed at a magnification of 30000X using a JEOL 1200EX electron microscope. Image acquisition, quantification and analysis of data were conducted blind to experimental condition. CYFIP1 and FMRP immunoreactivity was evaluated by counting DAB-labeled axons, dendrites and spines over the total of counted items (413 for CYFIP1; 725 for FMRP) and represented the average of the percentages between two animals. No labeling was seen on any of the control tissue.

Computational studies. The CYFIP1 structure extracted from the WRC crystal (PDB #3P8C) was

used for calculating the solvent-accessible surface (SAS) of the Lys743 (Naccess), the hydrophobic patches of CYFIP1-NCKAP1 (UCSF Chimera (Pettersen et al., 2004)), and as starting structure for the Molecular Dynamics (MD) Simulation (NAMD 2.8 package for GPU computing (Phillips et al., 2005), using the CHARMM force field (Brooks et al., 2009)). For the MD simulation, the CYFIP1 structure was immersed in a rectangular simulative box filled with 176.688 TIP3P explicit solvent molecules and rendered electro neutral by the introduction of two sodium counterions. The simulation was run for 135 ns after equilibration of the system. The analysis was performed with Gromacs 4.5.3 (Hess et al., 2008). CYFIP1 structure used as a receptor for docking simulations was derived from clustering of 135 ns MD simulation, while the eIF4E structure was extracted from the ternary eIF4E-m^7GpppA-4EBP1 complex (PDB #1WKW) (Tomoo et al., 2005). Docking simulations were performed using Haddock. All figures for the computational analysis were obtained using Pymol (The PyMOL Molecular Graphics System, Version 1.5.0.4 Schrödinger, LLC.) and UCSF Chimera (Pettersen et al., 2004).

m^7GTP chromatography. The procedure was slightly modified from (Napoli et al., 2008). Total mouse cortex was homogenized in ice-cold lysis buffer (100 mM NaCl, 10 mM $MgCl_2$, 10 mM Tris/HCl pH=7.5, 1% Triton X100, 1 mM DTT, 10 µg/ml Sigma protease inhibitor, 0.5 mM Na-orthovanadate, 10 mM β-glycerophosphate, 40 U/ml RNaseOUT^{TM}), and the homogenate was centrifuged 8 min at 12000 g at 4°C. m^7GTP-Sepharose beads (Amersham) were washed and incubated with 500 µg of extracts at 4°C for 90 min. In competition experiments, GppNHp- Rac1 (GTP-Rac1 analogue) or GDP-Rac1 was added during the incubation. The resin was washed with washing buffer and the proteins eluted with Laemmli buffer.

PAK1 PBD affinity chromatography. Cortical cells were extracted with magnesium-containing lysis buffer (MLB, 25 mM HEPES pH=7.5, 150 mM NaCl, 1% Triton X100, 10% glycerol, 10 mM $MgCl_2$, 1 mM EDTA, 10 µg/ml Sigma protease inhibitor and 40 U/ml RNasin). The lysates were incubated at 4°C for 60 min with 10 µl PAK1 PBD agarose beads (Cell Biolabs) in MLB. After three washes, proteins were eluted from the beads with 2.5X Laemmli buffer and analysed by immuno-blotting.

HEK293T cells. HEK293T cells were cultured in DMEM/F12 (Gibco) supplemented with 10% FBS. In serum starvation experiments, cells were deprived of FBS for 17 hrs and 10% FBS was added for 5, 10 or 60 min. Where indicated, 200 μM NSC23766 was applied for 20 min before cells collection.

YFP-CYFIP1 WT, Δ, H or E were transfected in HEK293T cells using Lipofectamine 2000^{TM} (Life Technologies). When indicated, *CYFIP1* was silenced with *CYFIP1* 3UTR-directed siRNAs as follows:

siRNA1:

5'-rCrCrC rUrUrU rArArC rArGrG rUrArC rArGrA rGrArU rArCrU rGrArA-3' 5'-rCrArG rUrArU rCrUrC rUrGrU rArCrC rUrGrU rUrArA rArGG G-3'

siRNA2:

5'-rCrArC rUrArA rArUrA rGrUrU rUrArC rGrGrA rGrArG rArArA rGrGrC-3' 5'-rCrUrU rUrCrU rCrUrC rCrGrU rArArA rCrUrA rUrUrU rArGT G-3'

A siRNA against the luciferase GL2 was used as scrambled control.
Transfected cells were collected after 48 hours, and proteins extracted in Laemmli buffer (Western blot) or in lysis buffer (IP).

DNA constructs. Human CYFIP1 coding region (Q7L576) was cloned into the pEYFP-C1 plasmid (Clontech) (YFP-CYFIP1) as previously described (Napoli et al., 2008). YFP-CYFIP1 mutant Δ, H and E were obtained by PCR site-directed mutagenesis as follows. MutΔ (CYFIP1 1-921) was obtained by replacing G922 with a stop codon and inserting a SpeI site. MutH was obtained by replacing A1003-I1010 with 8G. MutE was obtained as K725E (indicated in Napoli et al., 2008 as Lys743Glu). PCR products were then cloned into the HindIII and SpeI of pEYFP-CYFIP1. For FRET/FLIM, pEGFP-N1, pmCherry-C1, pByffu-mCherry-EGFP were used as reference/control. Human CYFIP1 coding region was cloned into XhoI of pByffu-mCherry-EGFP (mCherry-CYFIP1-EGFP). All plasmids were verified by sequencing.

Lentiviral *Cyfip1* silencing. Three independent validated shRNAs plasmids against mouse *Cyfip1* (# SHCLND-NM_011370 Sigma-Aldrich) were tested in neurons:

1) shRNA 315, targeting *Cyfip1* 3'UTR

5'- CCGGCCAGTAACTGATGGCATGTTTCTCGAGAAACATGCCATCAGTTACTGGTTTTTG - 3'

2) shRNA 318, targeting *Cyfip1* CDS

5'- CCGGCGCTGCTCTATCAGCCAAATTCTCGAGAATTTGGCTGATAGAGCAGCGTTTTTG - 3'

3) shRNA 319, targeting *Cyfip1* CDS

5'- CCGGCCAGATTCTCAACGATGAAATCTCGAGATTTCATCGTTGAGAATCTGGTTTTTG - 3'

shRNA 319 and 315 were used for subsequent experiments.

HEK293T cells were used as packaging cells and transfected by the calcium phosphate method (Chen and Okayama, 1987) with second generation plasmids (pLKO.1, Mission® shRNA, Sigma-Aldrich) (Naldini et al., 1996), carrying scrambled or *Cyfip1* shRNAs. Cell supernatants containing viruses carrying scrambled or *Cyfip1* shRNA plasmids were collected after 24 hr incubation and

filtered through 0.22 μm filters (Millipore). Virus-containing supernatants were added to DIV9 primary cortical neurons (1:5); after 5 h, the medium was changed and the silencing efficiency monitored at DIV15-16 by immuno-blotting.

Neuronal cell cultures and BDNF stimulation. Primary mouse cortical neurons were prepared as previously described (Ferrari et al., 2007). Neurons were stimulated with 100 ng/ml BDNF (Alomone Labs) for 30 min, with or without 10 min pre-treatment with 200 µM NSC23766 (Tocris Bioscience). When indicated, BDNF was applied together with 60 µM Cycloheximide (Sigma), 1 μg/ml Actinomycin D (Sigma) or 80 µM Dynasore (Sigma). Vehicle-treated neurons were used as control. Drugs were washed out and 5' later neurons were collected. Total protein extracts were prepared by extracting with 2.5X Laemmli buffer and analyzed by immuno-blotting. Alternatively, neurons were fixed and processed for immunofluorescence.

Synaptoneurosomes purification and stimulation. Cortical synaptoneurosomes were prepared as previously described (Napoli et al., 2008). Synaptoneurosomes were resuspended in HEPES-Krebs buffer (20 mM HEPES pH=7.4, 147 mM NaCl, 3 mM KCl, 10 mM glucose, 2 mM $MgSO_4$ and 2 mM $CaCl_2$), equilibrated at 37°C for 15 min, and then incubated for 30 min with 100 ng/mL BDNF (Alomone Labs) with or without pre-treatment of 10 min with 200 µM NSC23766 (Tocris Bioscience). Vehicle-treated synaptoneurosomes were used as control.

Immunofluorescence (IF). Primary cortical neurons were fixed with 4% paraformaldehyde (PFA), permeabilized with 0.2% Triton X-100 and incubated overnight with anti-CYFIP1 (1:200, mouse IgG1, Synaptic System), anti-eIF4E (1:100, mouse IgG2b, Santa Cruz), anti-NCKAP1 (1:100, rabbit, Sigma) or 1 h with anti-ARC (1:300, mouse, Synaptic Systems) or TRITC-phalloidin (1:1000, Sigma). After three washes, the specimens were incubated with following secondary antibodies: Alexa-488 anti-mouse IgG1 (1:1000, Invitrogen), Alexa-568 anti-mouse IgG2b (1:1000, Invitrogen), Cy5 anti-rabbit (1:500, Millipore). Staining with secondary antibodies only or unmatched secondary antibodies showed no signal.

The measurements for the co-localization studies were performed on a single focal plan (0.2 μm resolution). Background correction was performed selecting a "region-of-interest" (ROI) outside the neuron. Corrected images were then used to select the outlined 20 μm-segments of randomly chosen distal dendrites (with a distance from the cell body > 50μm). Quantitative measurements were then calculated using JaCoP plug-in of ImageJ. 20 μm segments starting at least 25 μm from the cell soma were analysed for at least 10 neurons/condition.

Measurements to detect ARC and F-actin were performed only in the dendritic spines outlined by F-GFP. Dendritic spines were tracked as ROI and fluorescence intensity measured only in the ROI. At least 150 spines for ARC and 50 spines for F-actin from two independent cultures were imaged and quantified.

Immunoprecipitation (IP). Immunoprecipitation was performed as previously described (Napoli et al., 2008). IPs with RNase-treated extracts were performed by adding 1.25 µg RNase A and 1000U RNaseT1 (Fermentas) during the overnight incubation. Proteins were extracted and analyzed for immunoblotting or mass spectrometry (LC-MS/MS).

FRET/FLIM. HEK293T cells were transfected with 500 ng of DNA using Lipofectamine 2000™ (Life Technologies). Cells were grown in DMEM/F12 for 24 hours, serum starved for 17 hours. When indicated, cells were treated with 200 µM NSC23766 for 20 min. FRET/FLIM measurements were performed as in (Sainlos et al., 2011). Specifically, in HEK293T FLIM was performed with the Time-Correlated Single Photon Counting (TCSPC) method on a multiphoton SP2 AOBS Leica confocal system using a HC Plan Apo CS 63X oil NA 1.32 objective (Leica Microsystems, Mannheim, Germany). The pulsed light source was a tunable Ti:Sapphire laser (Chameleon, Coherent Laser Group, Santa Clara, CA, USA). The laser was used at 900 nm in order to excite GFP. The laser repetition frequency was 80 Mhz which gave a 13 ns temporal window for lifetime measurements. The system was equipped with the TCSPC from Becker and Hickl (Berlin, Germany) comprising a PMC-100 detector characterized by a transit time spread of approximately 150 ps and SPC 830 photon counting and timing electronic card. A 510/40 band-pass filter (Chroma Technology, Rockingham VT, USA) was used to specifically detect the donor fluorescence. Fluorescence decay curves were obtained using single spot mode of SPCM software (Becker and Hickl). The mean lifetime was obtained from the exponential fit of the decay curve of the donor (EGFP) alone or of the donor in the presence of the acceptor (m-Cherry).

FRET/FLIM experiments in neurons were performed using a Leica DMI6000 microscope (Leica Microsystems, Wetzlar, Germany) and the HCX PL Apo 100X oil NA 1.4 objective as described in (Zhang et al., 2013). This microscope was equipped with the LIFA fluorescence lifetime attachment (Lambert Instrument, Roden, The Netherlands), which allows the generation of lifetime images by using the frequency domain method. This system consisted of a modulated intensified CCD camera Li^2 CAM MD, a modulated light excitation light source, a modulated GenIII image intensifier. GFP was excited using a modulated LED (Light-Emitted-Diode) at 477 nm (3W). Both the LED and the intensifier are modulated at frequency up to 100 MHz. A series of 12 images was recorded for each sample. By varying the phase shifts (12 times) between the illuminator and the intensifier modulation, the phase and modulation for each pixel of the image was calculated.

Consequently, the sample fluorescence lifetime image was determined using the manufacturer's software LI-FLIM software. Lifetimes were referenced to a 1 mM solution of fluorescein in saline (pH=10) that was set at 4.00 ns lifetime.

Time lapse. Embryonic rat hippocampal neurons (DIV9) were transfected with pEYFP-CYFIP1, pmCherry-eIF4E and pCerulean-NCKAP1 using Lipofectamine 2000™ (Life Technologies). Neurons were imaged 16-24h after transfection using a Leica SP5 confocal scan head mounted on

a DM 6000CS upright Leica microscope, equipped with a 63× 1.4 NA oil-immersion lens. Time-lapse images were acquired each 10 sec over 10 min after 10 min of BDNF stimulation with a resolution of 1024x512 pixels. Images were analyzed using Image J software (1.44p version). The average fluorescence intensity was measured by drawing a region of interest (ROI) of 500nm diameter that comprised the entire CYFIP1 punctum in the spine. Average intensity of each channel over time was measured in each ROI. Intensity values were normalized by the maximum intensity value in each channel. Normalized intensity values corresponding to NCKAP1 and eIF4E were divided by CYFIP1 intensity values in each time-lapse point and plotted against time.
Correlation between time and normalized intensity values was calculated by Pearson's coefficient in three independent spines. For thumbnails, original images were deconvolved using the 'Iterative Deconvolution' plugin in Image J.

Blue Native gel electrophoresis. Mouse brain cortices were homogenized in 3 ml lysis buffer (50 mM NaCl, 50 mM TrisHCl 7.4, 10 μl/ml protease inhibitors, 10 μl/ml phosphatases inhibitors and 40U/ml RNase OUT with/without detergent (0.2% Triton X-100)). The lysates were centrifuged 5 min at 1000g at 4°C, and then 10 min at 10000g at 4°C. 20 μg of lysates were loaded onto a NativePAGE 3-12% Bis-Tris Gel (Life Technologies) according to the manufacturer's indications and transferred in 1X TGS buffer (Bio-Rad) +20% MetOH on PVDF membrane. The membrane was destained in 10% acetic acid, 50% methanol for 10 min prior incubation with antibodies.

Diolistic staining of ex vivo brain slices. Mice were deeply anaesthetized with ketamine/xilazine mixture and transcardially perfused with paraformaldehyde 4% in PBS 1X. Brains were then excised and post-fixed in 4% paraformaldehyde. Vibratome sections of 150 µm at the hippocampal level were collected and preserved free floating in PBS 1X-sodium azide 0.02%. Diolistic staining was performed using a gene gun system (Helios Gene Gun System, Bio-Rad), as previously reported (Seabold et al., 2010). Briefly, bullets were prepared coating 100 mg of 1 µm Ø tungsten beads with 3 mg of DiI (1-1'-Dioctadecyl-3,3,3',3'- tetramethylindocarbocyanine perchlorate) (Sigma) both dissolved in methylene chloride (Sigma) over a glass slide. Beads were used to prepare about 50 bullet tubes (final concentration of about 0.06 mg DiI/2 mg of tungsten beads for each bullet). Tungsten beads coated with DiI were then shot over slices. Shooting was performed twice for each slice, using a 3 μm pore paper filter, at a helium pressure of 150 p.s.i. and at a distance of about 4-5 cm from the slice. Treated slices were left free floating in PBS 1X at 4°C O.N., then mounted over glass slides and cover-slipped with fluorescence anti-fading aqueous mounting media (Sigma, St Louis, MO, USA). Neurons of layers I-III of the frontal cortex from 3 animals per group were analyzed.

Disease annotation based on published literature. Genes were screened for their involvement in diseases by manually interrogating OMIM, PubMed, SFARI Gene, AutismKB, SZGene and AlzGene. Information based on genome-wide association studies (GWAS), genome-wide CNV

studies, high and low-scale expression profiling and low-scale gene studies were screened and only direct evidence of diseases involvement were reported.

Gene–based analyses. Meta-analytic p-values obtained from the Schizophrenia Psychiatric Genome-Wide Association Study Consortium (Ripke et al., 2011) were used as input to VEGAS software (Liu et al., 2010) to determine gene-based p-values the meta-analysis included 9,394 cases and 12,462 controls from 17 samples from the Stage 1 analysis of SPGWAS. Data cleaning was performed as previously described (Ripke et al., 2011). Briefly, each sample was investigated by using Principal Components (PC) population structure analysis. Imputation was performed with CEU+TSI HapMap Phase 3 data (UCSC hg18/NCBI 36), and association testing for imputed SNP allele dosages was performed using standard logistic regression with study indicator and PCs as covariates. All single studies were analysed separately followed by an SE-weighted meta-analysis. The meta-analytic SNP p-values obtained in the SPGWAS study (Stage 1 analysis on 17 samples) were used as input for the current gene-based tests. We examined 2995 SNPs in 36 genes (±50 kb) that are part of the CYFIP1 interactome for association with schizophrenia, using VEGAS (version 0.8.27) (Liu et al., 2010). We ran VEGAS using the option top 10%, which implies that only the top 10% of SNPs in a gene are considered for the gene-based association test. The 'top 10%" option in VEGAS is generally more powerful than the default including all SNPs in a gene. The software applies a test that incorporates information from a set of markers within a gene (or region) and accounts for LD between markers by using adaptive simulations from the multivariate normal distribution. An empirical p-values of 0 from 100000 simulations can be interpreted as $p < 10$ E-5, which exceeds a Bonferroni-corrected threshold of $p < 0.00139$ [~0.05/36 (number of tested autosomal genes)

Supplemental References

Brooks, B.R., Brooks, C.L., 3rd, Mackerell, A.D., Jr., Nilsson, L., Petrella, R.J., Roux, B., Won, Y., Archontis, G., Bartels, C., Boresch, S., *et al.* (2009). CHARMM: the biomolecular simulation program. Journal of computational chemistry *30*, 1545-1614.

Ferrari, F., Mercaldo, V., Piccoli, G., Sala, C., Cannata, S., Achsel, T., and Bagni, C. (2007). The fragile X mental retardation protein-RNP granules show an mGluR-dependent localization in the post-synaptic spines. Mol Cell Neurosci *34*, 343-354.

Hess, B., Kutzner, C., van der Spoel, D., and Lindahl, E. (2008). GROMACS 4: Algorithms for highly efficient, load-balanced, and scalable molecular simulation. J Chem Theory Comput *4*, 435-447.

Liu, J.Z., McRae, A.F., Nyholt, D.R., Medland, S.E., Wray, N.R., Brown, K.M., Hayward, N.K., Montgomery, G.W., Visscher, P.M., Martin, N.G., *et al.* (2010). A versatile gene-based test for genome-wide association studies. American journal of human genetics *87*, 139-145.

Mohr, E., and Richter, D. (2003). Molecular determinants and physiological relevance of extrasomatic RNA localization in neurons. Front Neuroendocrinol *24*, 128-139.

Napoli, I., Mercaldo, V., Boyl, P.P., Eleuteri, B., Zalfa, F., De Rubeis, S., Di Marino, D., Mohr, E., Massimi, M., Falconi, M., *et al.* (2008). The fragile X syndrome protein represses activity-dependent translation through CYFIP1, a new 4E-BP. Cell *134*, 1042-1054.

Ostroff, L.E., Cain, C.K., Bedont, J., Monfils, M.H., and Ledoux, J.E. (2010). Fear and safety learning differentially affect synapse size and dendritic translation in the lateral amygdala. Proc Natl Acad Sci U S A *107*, 9418-9423.

Pettersen, E.F., Goddard, T.D., Huang, C.C., Couch, G.S., Greenblatt, D.M., Meng, E.C., and Ferrin, T.E. (2004). UCSF Chimera--a visualization system for exploratory research and analysis. Journal of computational chemistry *25*, 1605-1612.

Phillips, G.R., Huang, J.K., Wang, Y., Tanaka, H., Shapiro, L., Zhang, W., Shan, W.S., Arndt, K., Frank, M., Gordon, R.E., *et al.* (2001). The presynaptic particle web: ultrastructure, composition, dissolution, and reconstitution. Neuron *32*, 63-77.

Phillips, J.C., Braun, R., Wang, W., Gumbart, J., Tajkhorshid, E., Villa, E., Chipot, C., Skeel, R.D., Kale, L., and Schulten, K. (2005). Scalable molecular dynamics with NAMD. Journal of computational chemistry *26*, 1781-1802.

Ripke, S., Sanders, A.R., Kendler, K.S., Levinson, D.F., Sklar, P., Holmans, P.A., Lin, D.Y., Duan, J., Ophoff, R.A., Andreassen, O.A., *et al.* (2011). Genome-wide association study identifies five new schizophrenia loci. Nat Genet *43*, 969-976.

Sainlos, M., Tigaret, C., Poujol, C., Olivier, N.B., Bard, L., Breillat, C., Thiolon, K., Choquet, D., and Imperiali, B. (2011). Biomimetic divalent ligands for the acute disruption of synaptic AMPAR stabilization. Nature chemical biology *7*, 81-91.

Seabold, G.K., Daunais, J.B., Rau, A., Grant, K.A., and Alvarez, V.A. (2010). DiOLISTIC labeling of neurons from rodent and non-human primate brain slices. J Vis Exp.

Tomoo, K., Matsushita, Y., Fujisaki, H., Abiko, F., Shen, X., Taniguchi, T., Miyagawa, H., Kitamura, K., Miura, K., and Ishida, T. (2005). Structural basis for mRNA Cap-Binding regulation of eukaryotic initiation factor 4E by 4E-binding protein, studied by spectroscopic, X-ray crystal structural, and molecular dynamics simulation methods. Biochim Biophys Acta *1753*, 191-208.
